# A novel unbiased measure for motif co-occurrence predicts combinatorial regulation of transcription

**DOI:** 10.1186/1471-2164-13-S7-S11

**Published:** 2012-12-07

**Authors:** Alexis Vandenbon, Yutaro Kumagai, Shizuo Akira, Daron M Standley

**Affiliations:** 1 Laboratory of Systems Immunology, Immunology Frontier Research Center, Osaka University, 3-1 Yamada-oka, Suita, Osaka 565-0871, Japan; 2 Laboratory of Host Defense, Immunology Frontier Research Center, Osaka University, 3-1 Yamada-oka, Suita, Osaka 565-0871, Japan; 3 Department of Host Defense, Research Institute for Microbial Diseases, Osaka University, 3-1 Yamada-oka, Suita, Osaka 565-0871, Japan

## Abstract

**Background:**

Multiple transcription factors (TFs) are involved in the generation of gene expression patterns, such as tissue-specific gene expression and pleiotropic immune responses. However, how combinations of TFs orchestrate diverse gene expression patterns is poorly understood. Here we propose a new measure for regulatory motif co-occurrence and a new methodology to systematically identify TF pairs significantly co-occurring in a set of promoter sequences.

**Results:**

Initial analyses suggest that non-CpG promoters have a higher potential for combinatorial regulation than CpG island-associated promoters, and that co-occurrences are strongly influenced by motif similarity. We applied our method to large-scale gene expression data from various tissues, and showed how our measure for motif co-occurrence is not biased by motif over-representation. Our method identified, amongst others, the binding motifs of HNF1 and FOXP1 to be significantly co-occurring in promoters of liver/kidney specific genes. Binding sites tend to be positioned proximally to each other, suggesting interactions exist between this pair of transcription factors. Moreover, the binding sites of several TFs were found to co-occur with NF-κB and IRF sites in sets of genes with similar expression patterns in dendritic cells after Toll-like receptor stimulation. Of these, we experimentally verified that CCAAT enhancer binding protein alpha positively regulates its target promoters synergistically with NF-κB.

**Conclusions:**

Both computational and experimental results indicate that the proposed method can clarify TF interactions that could not be observed by currently available prediction methods.

## Background

Gene expression in multicellular eukaryotes varies considerably between tissues and can change dramatically even within the same cell type. Regulation of transcription is one of the fundamental mechanisms for controlling the observed diversity in gene expression [1,2], and recent studies have underscored the importance of combinatorial regulation by multiple transcription factors (TFs) in this regard [3-6]. Progress is also being made towards experimental methods for testing combinatorial regulators on a larger scale in near-physiological conditions [7]. Combinatorial regulation can explain, in general, how a relatively small number of TFs can govern gene expression under diverse conditions.

One such example is the regulation of gene expression in immune responses. Pathogen recognition in the vertebrate innate immune system is initially performed by a limited number of pattern-recognition receptors (PRRs). The Toll-like receptors (TLRs) are a family of PRRs responsible for the recognition of a wide variety of pathogen-associated ligands, such as lipopolysaccharide, viral RNA, unmethylated CpG DNA and so on. The recognition of ligands activates signaling pathways leading to the activation of several TFs, such as NF-κB, and IRFs. These TFs are known to induce expression of various genes and evoke pleiotropic immune responses. Although several studies have addressed the importance of combinatorial transcriptional regulation in TLR signaling [8,9], little is known about which specific combinations of TFs are involved.

Various bioinformatics strategies have been used for the prediction of cooperation between TFs. Some studies have used a combination of features, such as co-expression data and protein-protein interactions [10,11]. Other studies utilized a combination of chromatin immunoprecipitation combined with microarray (ChIP-chip) data and expression data [12-16]. However, while the integration of heterogeneous experimental data sources is potentially very powerful, in practice such data is too scarce to be of use to a particular tissue of interest, especially for higher eukaryotes like humans and mice. ChIP-chip data, in particular, is available for a very limited number of TFs, in a limited number of cell types. In addition, in the case of *de novo *predicted regulatory motifs, it might not be known what protein (if any) is binding the motif in question, which restricts the applicability of ChIP-chip analysis. For these reasons, a number of studies have focused on identifying combinatorial regulation solely based on predicted transcription factor binding sites (TFBSs). For example, Murakami et al. used position weight matrices (PWMs) to predict TFBSs on a genomic scale in order to quantify the co-occurrence of regulatory motifs in human promoters [17]. Sudarsanam and colleagues used a cumulative hypergeometric distribution to predict regulatory motifs co-occurring on a genome-wide scale in yeast [18]. Other studies have described measures for co-occurrence of pairs of motifs as a measure to predict TF synergy [19]. Synthetic libraries of promoters have been used to study combinatorial regulation using thermodynamic models [20], and more recently, combinations of oligomers have been used to predict from sequence EP300-bound and CREBBP-bound enhancers in three mammalian cell types [21].

A small number of studies have attempted to identify pairs of co-occurring motifs in the promoters of co-expressed genes [22,23]. However, methods for predicting combinatorial regulation from predicted TFBSs are plagued by a number of problems. These include similarities beween the PWMs used to predict TFBSs, biases caused by motif over-representation, and difficulty of evaluating the significance of observed co-occurrences using standard statistical tests.

In this study, first we describe a new measure for TFBS pair co-occurrence. For each PWM pair (*A,B*), we calculate the frequency of motif *B *in sequences containing one or more *A *sites, as well as the frequency of motif *B *in sequences that lack *A *sites. We use the ratio of these two frequencies, the frequency ratio (*FR*), as a measure for co-occurrence. Applying this measure on the TFBSs in the genomic set of human and mouse promoters, we observed how co-occurrence tendencies are strikingly different between promoters with high GC content and CpG scores and promoters with low GC content and CpG scores, with the latter having a higher variety in *FR *values. We also observed a strong influence of TFBS GC content differences.

Based on the above observations, we developed a method for predicting co-regularing pairs of TFs in a set of co-expressed genes. Given the promoter sequences for a set of genes that are co-expressed, we identify motif pairs that co-occur more often than expected. We use the relative increase in co-occurence in the co-expressed set of genes as an indicator of combinatorial regulation.

Our proposed method was designed to overcome the problems associated with previously reported statistics-based measures of co-regulation. In order to obtain a measure of statistical significance, we compare the observed *FR *values for pairs of motifs in a set of co-expressed genes with those of sets of genes sampled at random, thus taking into account biases caused by genome-wide co-occurrence tendencies. We applied our approach to a number of sets of co-expressed mouse genes, and found a number of significantly co-occurring PWMs pairs. Importantly, the proposed approach was not biased by TFBS motif over-representation, and could thus detect co-occurrences missed by existing approaches. For the identified TF pair NF-κB - C/EBPαwe experimentally validated the co-regulation after TLR stimulation in dendritic cells. Since the proposed method does not rely on ChIP-chip data, it is generally applicable and can complement existing computational methods for discovery of TF co-regulation.

## Methods

We refer to Additional file 1 for a workflow of our framework for the detection of co-occurring motifs.

### Promoter sequences

We used a combination of DBTSS data [24], CAGE data [25], and annotation data from the UCSC Genome Browser [26] to define transcription start site (TSS) positions for both human and mouse genes, as described before [27]. The regions from -1000 to +200 were extracted from the repeat-masked hg18 and mm9 versions of the human and mouse genome. For each pair of highly similar sequences (BLAST E value < 1e-70, threshold decided after visual inspection of alignments) one sequence was removed from our sequence dataset in order to reduce biases caused by duplicated sequences.

### Position weight matrix dataset

From the TRANSFAC [28] and JASPAR [29] databases all vertebrate PWMs were extracted. Redundancies were removed using tomtom [30] by the following strategy: for each pair of similar PWMs (tomtom E value < 1, and overlap between motifs > 75% of each motifs length) the motif with the lowest information content was removed from our dataset. Pairs were considered in order of increasing tomtom E value. This resulted in a PWM dataset of 199 non-redundant PWMs, each representing a group of similar PWMs. For each PWM a score threshold was set in a way that there is about 1 hit per 5000 bps in the mouse promoter sequences. GC content values of PWMs were calculated as the average of the probability of nucleotides C and G over all positions of the PWMs.

### Measure for TFBS co-occurrence: frequency Ratio

As a measure of TFBS co-occurrence we introduce the Frequency Ratio (*FR*) value. Consider two TFs, TF *A *and TF *B*, whose binding preferences are represented by PWM *A *and PWM *B *respectively. Given a set of sequences and the predicted sites for both PWMs, we calculate the *FR(B|A)*, the tendency of sites for TF *B *to co-occur with those of TF *A*, as follows. First, we define *seq(A) *as the number of sequences containing at least one site for motif *A*, and *n(B|A) *as the number of sites for motif *B *co-occurring with one or more sites for motif *A*. From these we calculate *frequency(B|A)*, a measure for the number of *B *sites co-occurring with *A *sites:

(1) $$frequency\left(B|A\right)=n\left(B|A\right)/seq\left(A\right)$$

Likewise, we define *frequency(B|!A) *as the number of predicted sites for motif *B *per sequence lacking sites for motif *A*:

(2) $$frequency\left(B|!A\right)=n\left(B|!A\right)/seq\left(!A\right)$$

where *n(B|!A) *is the number of *B *sites in the set of promoters lacking *A *sites and *seq(!A) *is the number of sequences without *A *sites. We calculate the ratio of these two frequency values, *FR(B|A)*:

(3) $$FR\left(B|A\right)=\frac{\frac{n\left(B|A\right)}{seq\left(A\right)}}{\frac{n\left(B|!A\right)}{seq\left(!A\right)}}=\frac{number\;of\;B\;sites\;per\;sequence\;having\;\geq 1\;A\;site}{number\;of\;B\;sites\;per\;sequence\;having\;no\;A\;site}$$

*FR(B|A) *is a measure for the tendency of sites of motif *B *to be present in sequences having at least one *A *site compared to sequences not having an *A *site. In order to limit the bias caused by overlapping sites for pairs of similar motifs, sites for motif *B *overlapping *A *sites are not included in *n(B|A)*. In the case of homotypic motif pairs (where motif *B *and motif *A *are the same motif), there are obviously no motif *B *sites in sequences not containing sites for motif *A*. In this case we define *frequency(A|!A) *= 1. In this case *FR(A|A) *can be interpreted as the average number of *A *sites in sequences containing at least one *A *site. Note that the *FR *measure is not limited to TFBS motifs, but can be used for other sequence motifs and nucleotide oligomers.

### Micro-array gene expression data

We used micro-array expression data for a large number of human and mouse tissues [31], and for dendritic cells (DCs) after stimulation with a number of immune stimuli [9] (GSE17721). The raw intensity data were processed to calculate robust multi-array average (RMA) values. Genes with at least 3-fold differential expression between any pair of samples were picked up. Expression values for each gene were rescaled to mean 0 and standard deviation 1, and dimension reduction was performed with principal component analysis. The gene expression patterns were hierarchically clustered with Ward's algorithm and divided into subclusters. All analyses were performed using R and Bioconductor.

### Definition of CpG^high ^promoters and CpG^low ^promoters

We based our definition of CpG^high ^and CpG^low ^promoters on previously reported definitions for CpG islands [32]. For each promoter sequence we defined the ratio of each nucleotide as the number occurrences of that nucleotide divided by the number of non-masked nucleotides in the sequence. The GC content is then the sum of the C ratio and the G ratio. The CpG score is the observed ratio of CpG dinucleotides divided by the ratio expected from the ratio of C and G nucleotides. We defined "CpG^high ^promoters" as promoters with a GC content ≥ 50% and a CpG score ≥ 0.6. Promoters not meeting these requirements were labeled as "CpG^low ^promoters". These two conditions gave us 6750 CpG^high ^promoters in mouse (37% of total) and 9029 CpG^high ^promoters in human (50% of total).

### Detection of over-represented TFBSs

As a measure for over-representation of a TFBS motif in a set of sequences we use the Over-Representation Index (*ORI*) as defined by Bajic et al. [33]. P-values for *ORI *values were assigned by random sampling sets of sequences of the same size as the set of interest from the genomic set of promoter sequences. For the estimation of p-value in CpG^high ^promoters and CpG^low ^promoters the sampling was done from the genomic set of CpG^high ^promoters and the genomic set of CpG^low ^promoters, respectively. As p-value threshold for over-representation we used the threshold of 0.01.

### Co-occurrence significance in promoters of co-regulated genes

For each co-expressed set of genes containing at least 50 genes, we identified over-represented TFBSs, as described above. For each pair of motifs (*A,B*), where *A *is an over-represented motif and *B *is any of the 199 PWMs, we calculated *FR(B|A)_set _*in the promoter sequences corresponding to the set of co-expressed genes. The significance of *FR(B|A)_set _*was evaluated using a sampling approach. In this sampling approach, a set of sequences are sampled from the genomic set of sequences until the same number of *B *sites and *A *sites as in the co-expressed cluster is obtained. From these, a *FR(B|A)_sampled _*is calculated, reflecting the genome wide co-occurrence tendencies of the pair of motifs. This is repeated a large number of times (10^5 ^times in this study), and the p-value of *FR(B|A)_set _*is defined as the ratio of the number of times where *FR(B|A)_sampled _*≥ *FR(B|A)_set_*.

### Generation of artificial and semi-artificial promoter sequences

Artificial promoter sequences were constructed by generating sequences of the same length as the actual promoter sequences used in this study, where at each position the nucleotide is decided using a uniform distribution over the alphabet (A,C,G,T). Semi-artificial sequences were generated by scanning through actual sequences and randomly adding either a G or C to the semi-artificial sequence when a G or C was encountered in the actual sequence; and randomly adding either an A or T when an A or T was encountered.

### Construction of plasmids, transfection, and luciferase assay

Promoter sequences of selected genes were PCR amplified and cloned into pGL3-basic vectors (Promega). Sequences from about -1200 to +250 relative to transcription start site were cloned. kB-tandem reporters were purchased from Promega. Complementary DNA for TFs was PCR amplified and cloned into pEF-BOS expression vectors. The resulting reporter plasmids and TF over-expression plasmids were co-transfected into HEK 293 cells with pRL-TK encoding *Renilla *luciferase (Promega) and appropriate signaling molecules with using Lipofectamin 2000 (Invitrogen). At 24 hours after transfection, the cells were lysed and subjected to reporter assay according to the manufacture's instruction (Promega). The primers used will be provided upon request.

## Results and discussion

### Frequency ratio, a novel measure for co-occurrence of two TFBSs: general results and genomic tendencies

As a measure for the co-occurrence of the TFBSs for two TFs, TF *A *and TF *B*, we propose the Frequency Ratio, *FR *(see Methods section). The *FR(B|A) *value is a measure for the tendency of motif *B *to co-occur with motif *A*. On a molecular level, it reflects the tendency of TF *B *to bind the same promoters as TF *A*, although this does not necessarily imply a direct physical interaction between *A *and *B*. Cases where *FR(B|A) *values are higher than 1 reflect a tendency of TF *B *to bind promoter sequences that also are bound by TF *A*, while *FR(B|A) *values lower than 1 reflect a tendency for TF *B *to bind to promoter sequences not bound by TF *A*. To avoid biases caused by motif similarities, sites where motifs *A *and *B *overlap were discarded before the calculation of the frequency values. Note that *FR(B|A) *is not necessarily the same as, or similar to, *FR(A|B) *(Supporting text in Additional file 2).

**Figure 1:**
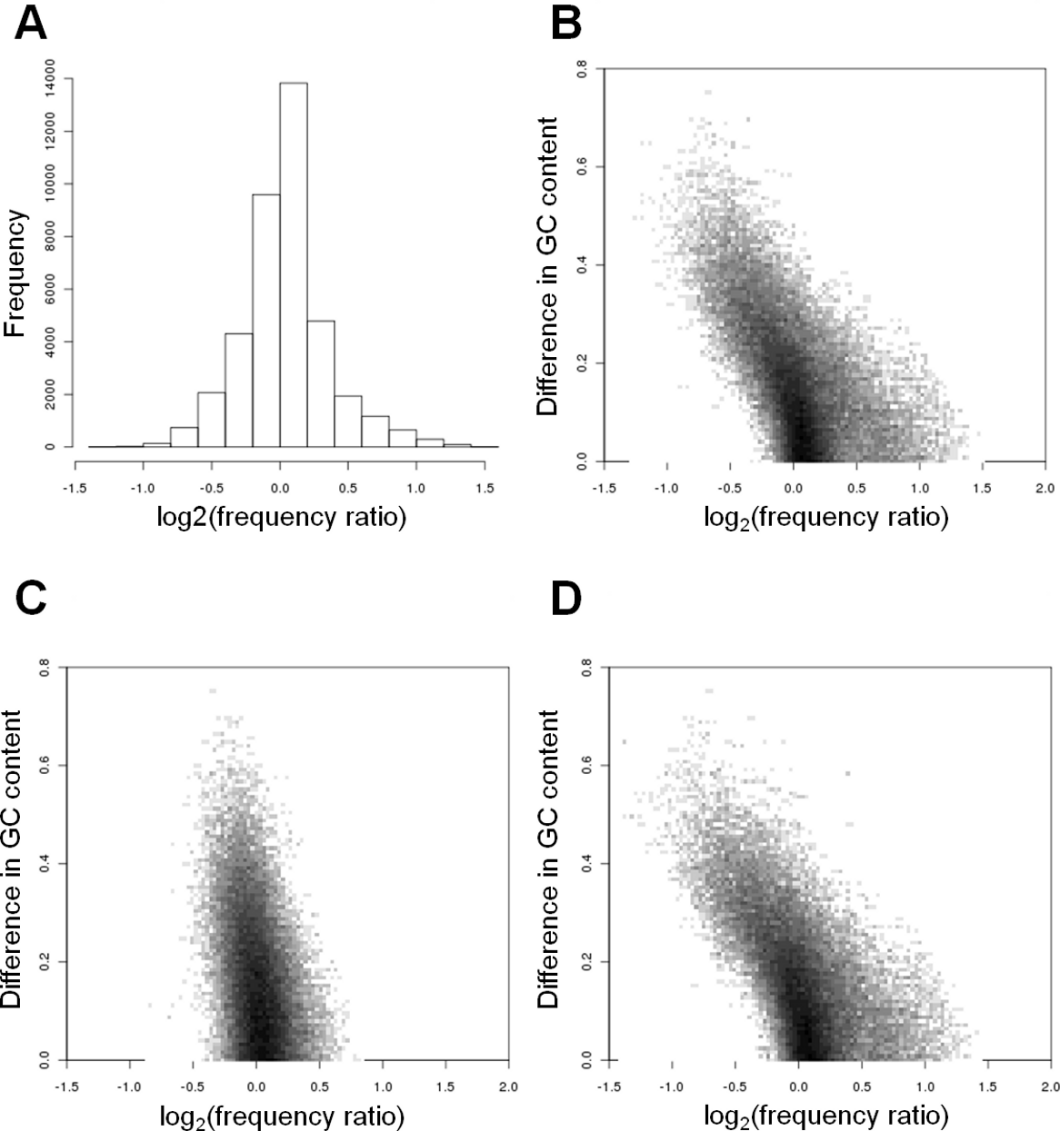
**Genome-wide tendencies of Frequency Ratios**. (A) Histogram of *FR *values for all PWM pairs in the genomic set of mouse promoter sequences. (B,C,D) Plots of GC content differences as a measure of PWM-to-PWM dissimilarity (Y-axis) versus *FR *values (X-axis, same as in A), for all promoters (B), CpG^high ^promoters (C), and CpG^low ^promoters (D).

Using the above definition of *FR *we calculated the genome-wide *FR *values for all 39,601 (199 × 199) TFBS motif pairs, in the genome-wide sets of 18,218 human promoter sequences, and 18,168 mouse promoter sequences. A histogram of *FR *values in the genomic set of mouse promoters is shown in Fig. 1A. Although the majority of PWM pairs have *FR *values close to 1 (84.9% of the pairs have a *FR *value between 0.7 and 1.3), some pairs have high or low *FR *values. Similar observations were made for human sequences (Fig. S2A in Additional file 3). The outliers with large or small *FR *values indicate the genome-wide tendencies for high or low co-occurrence of sequence motifs, respectively. These genome-wide tendencies represent reference values to which we will compare the *FR *values of particular sets of co-expressed genes.

### Similar sequence motif pairs tend to be co-occurring

Next, we analyzed the correlation between *FR *values and motif-motif similarity. We used the difference of GC content between pairs of motifs as an indicator of motif similarity. Fig. 1B shows a plot of the difference in GC content between pairs of motifs versus *FR *for the genomic set of mouse promoter sequences. This figure clearly shows that motif pairs with a smaller difference in GC content tend to have higher *FR *values, while motif pairs with different GC content tend to have lower *FR *values. A similar tendency was obtained in human promoter sequences (Fig. S2B in Additional file 3). Since we excluded overlapping sites, the tendency of these motifs to co-occur cannot be explained simply by a tendency of sites for similar PWMs to overlap with each other. In semi-artificial promoter sequences, where overall GC content and local GC content fluctuations were identical to those of real promoter sequences, a similar tendency was observed (84.7% of the pairs had a *FR *value between 0.7 and 1.3; Fig. S3A in Additional file 4). On the other hand, in completely artificial sequences with 50% GC content, this tendency was not observed: the vast majority of motif pairs had *FR *values close to 1 (99.0% of the pairs had a *FR *value between 0.7 and 1.3; Fig. S3B in Additional file 4).

### CpG^low ^promoters have a higher variety of *FR *values than CpG^high ^promoters

Given the observed influence of GC content on co-occurrence, we decided to separately investigate the tendencies in CpG^high ^and CpG^low ^promoters. The genome-wide set of promoter sequences was divided into a set of CpG^high ^promoters and CpG^low ^promoters (see Methods section), and the *FR *values in each set were calculated. Figure 1C shows the difference in GC content between pairs of motifs versus their *FR *values in the genome-wide CpG^high ^promoter set in mouse. From this figure we can see that for the CpG^high ^promoter set, the tendency for motif pairs with a smaller (larger) difference in GC content to have higher (lower) *FR *values was not observed. In contrast, for the CpG^low ^promoters (Fig. 1D) such a tendency was clearly observed. These trends were also found in human sequences (Fig. S2C,D in Additional file 3) and semi-artificial promoters sequences (Fig. S3C,D in Additional file 4). Assuming that the variety in *FR *values reflects the potential of sequences to encode combinatorial regulation, these results suggest that the regulatory complexity of CpG^low ^promoters is higher than that of CpG^high ^promoters.

An additional result supporting the notion that CpG^low ^promoters have a higher potential for combinatorial regulation was obtained from the analysis of *FR *values of 200 randomly selected oligomers. Although these oligomers are likely not to be associated with any regulatory motifs, the tendencies of *FR *values are similar to those we observed for PWM motifs in CpG^high ^and CpG^low ^sequences (Fig. S4 in Additional file 5).

### Only few motif pairs have high or low FR values on a genome-wide level

The above observations raise the question to what extent genome-wide *FR *values are indicative of combinatorial regulation between pairs of TFs on a genome-wide level. We compared *FR *values observed in the genome-wide set of promoter sequences with those observed in 10 sets of semi-artificial sequences. To take into account the influence of GC content difference, we divided pairs of PWMs into 10 bins according to their pairwise GC content difference. Table 1 shows all pairs of PWMs with high and low tendencies to co-occur on a genome-wide scale (*FR *values higher/lower than 99.99% of the *FR *values observed for PWM pairs with similar GC content differences in semi-artificial sequences).

**Table 1:** PWM pairs with high and low *FR *values in the genomic set of promoters.

Transcription factor A (PWM ID)	Transcription factor B (PWM ID)	*FR_genomic_*(B|A) (high/low) *	Species	GC content difference
TBP (M00471)	Six6 (PB0163)	2.75 (high)	human	0.28
Six6 (PB0163)	TBP (M00471)	2.56 (high)	human	0.28
POU1F1, POU3F2 (M00463)	Six6 (PB0163)	2.10 (high)	mouse	0.22
TBP (M00471)	POU2F1, Sox15 (M00162)	2.09 (high)	mouse	0.21
POU2F1, Sox15 (M00162)	TBP (M00471)	2.08 (high)	mouse	0.21
TBP (M00471)	Cux1 (PH0017)	2.07 (high)	mouse	0.22
Six6 (PB0163)	POU1F1, POU3F2 (M00463)	2.06 (high)	mouse	0.22
Six6 (PB0163)	TBP (M00471)	1.89 (high)	mouse	0.28
various homeobox TFs (PH0077)	TBP (M00471)	1.88 (high)	mouse	0.27
Sox17, Sox8 (PB0178)	TBP (M00471)	1.80 (high)	mouse	0.28
Zfp105 (PB0197)	TBP (M00471)	1.80 (high)	mouse	0.29
TBP (M00471)	Six6 (PB0163)	1.79 (high)	mouse	0.28
TBP (M00471)	Sox17, Sox8 (PB0178)	1.79 (high)	mouse	0.28
TBP (M00471)	various homeobox TFs (PH0077)	1.76 (high)	mouse	0.27
TBP (M00471)	Zfp105 (PB0197)	1.75 (high)	mouse	0.29
C/EBP factors (M00201)	TBP (M00471)	1.53 (high)	mouse	0.34
MITF-TFE family (bHLH) (M01029)	E2F TFs (M00516)	0.65 (low)	mouse	0.12
E2F TFs (PB0009)	MITF-TFE family (bHLH) (M01029)	0.54 (low)	mouse	0.15

* (high/low) indicates whether the genomic *FR *is higher or lower than expected.

Two important observations can be made. The first point is that out of the 39,601 pairs of PWMs, only very few have exceptionally high/low *FR *values (Table 1). In mouse promoters 14 pairs, and in human promoters 2 pairs of PWMs had exceptionally high values, while in mouse promoters 2 pairs had low values. In human and mouse CpG^high ^sequences and mouse CpG^low ^sequences, no pairs with exceptional values were found. These indicate that the vast majority of *FR *values in true sequences are within the range of values we can expect to find in semi-artificial sequences lacking any biological meaning. A second point is that, interestingly, most of the pairs with high values involve the TBP motif (TATA box), a core promoter motif. This motif is thought to be typically present in non-CpG island-associated promoters of genes with relatively strictly regulated transcription initiation. On the other hand, we found the GC-rich and CpG-rich E2F TF motif, which might be indicative of CpG-rich sequences, to have low co-occurrence with a regulatory motif (TFE).

In conclusion, the above observations support the hypothesis that the genome-wide variation in *FR *values, except for those involving a small number of exceptional sequence motifs such as the TATA box and GC-box, is mainly a result of sequence variations, and not an indication of genome-wide combinatorial interactions between TFs. It is important that these genome-wide biases are taken into account by approaches that predict combinatorial regulation in smaller sets of co-regulated genes.

### The *FR *approach allows for detection of co-occurring regulatory motifs in tissue-specific promoter sequences without bias caused by TFBS over-representation

Next, we turned our attention to the problem of finding significantly co-occurring motifs in the promoter sequences of sets of co-expressed genes. We used gene expression data for a large number of mouse tissues [31] to define clusters of co-expressed genes (Fig. S5 in Additional file 6, Table S1 in Additional file 7), and applied our approach to each cluster containing at least 50 genes (see Methods). Below, we present some of our findings for a set of 155 genes with high expression in mouse liver and kidney. Of these, only 13 genes were associated with a CpG^high ^promoter. In the following discussion we focused on the CpG^low ^promoters. The most over-represented motifs in this set of promoters were those for PWMs for HNF1, HNF4, and a number of nuclear receptors (Table S2 in Additional file 8). These factors and their importance in liver-specific regulation of transcription have been widely reported [34-37].

**Figure 2:**
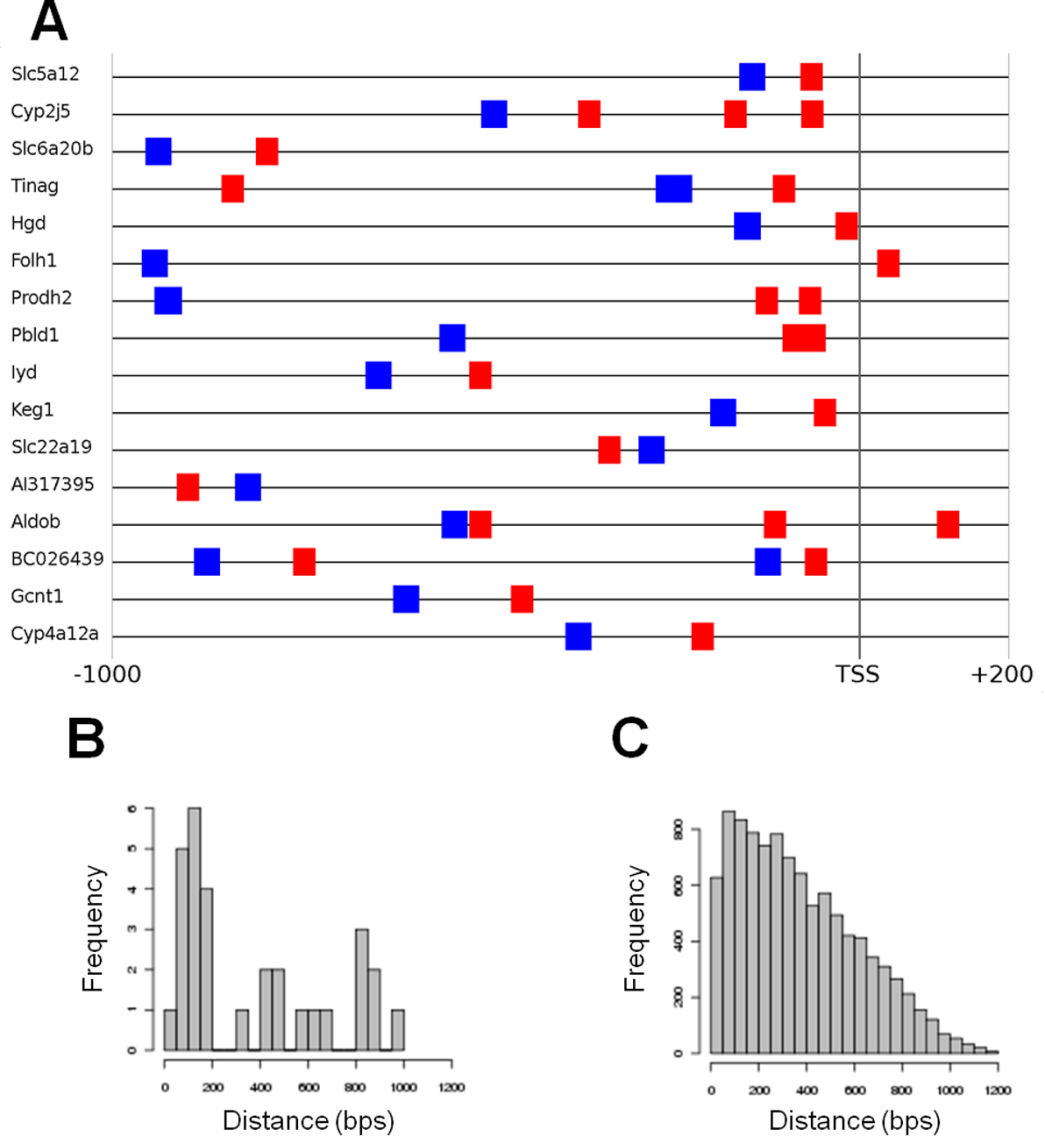
**Predicted HNF1 and FOXP1 binding sites in the promoters of liver- and kidney-specific genes**. (A) Visual representation of predicted TFBSs for HNF1 (green) and FOXP1 (red) in promoters containing at least 1 predicted site for each TF. Gene symbols are indicated at the left. (B) Histogram of distances between pairs of HNF1 and FOXP1 sites in the promoters of liver- and kidney-specific genes. (C) Histogram of distances between pairs of HNF1 and FOXP1 sites in the genomic set of promoters.

As described in Methods, the significance of co-occurrence for each *A*-*B *TF pair present in the set of sequences was estimated by sampling sequences from the genomic set of sequences until the same number of *A *and *B *sites were obtained, and the p-value of *FR(B|A)_set _*was computed. In the kidney/liver-specific set we found 11 co-occurrences with p-values < 0.01. In one case, the PWM for FOXP1 co-occurred significantly with HNF1 sites. FOXP1 plays a role in the development of various organs, including liver. In this set of promoters, 17 out of 21 non-overlapping FOXP1 sites co-occurred with HNF1 sites, yielding a *FR *value of 5.5 (Fig. 2A). Given the genomic *FR *value (1.6), we would expect only about 10.7 FOXP1 sites to co-occur with HNF1 sites on average (see Methods, from Eq. 3). Moreover, the distance between FOXP1 sites and HNF1 sites was biased towards proximal positioning: of the 26 FOXP1-HNF1 site pairs, 14 were separated by less than 200 bps (Fig. 2B,C). In addition, visual inspection of the site pairs revealed a preference of the FOXP1 site to be upstream of the HNF1 site.

**Figure 3:**
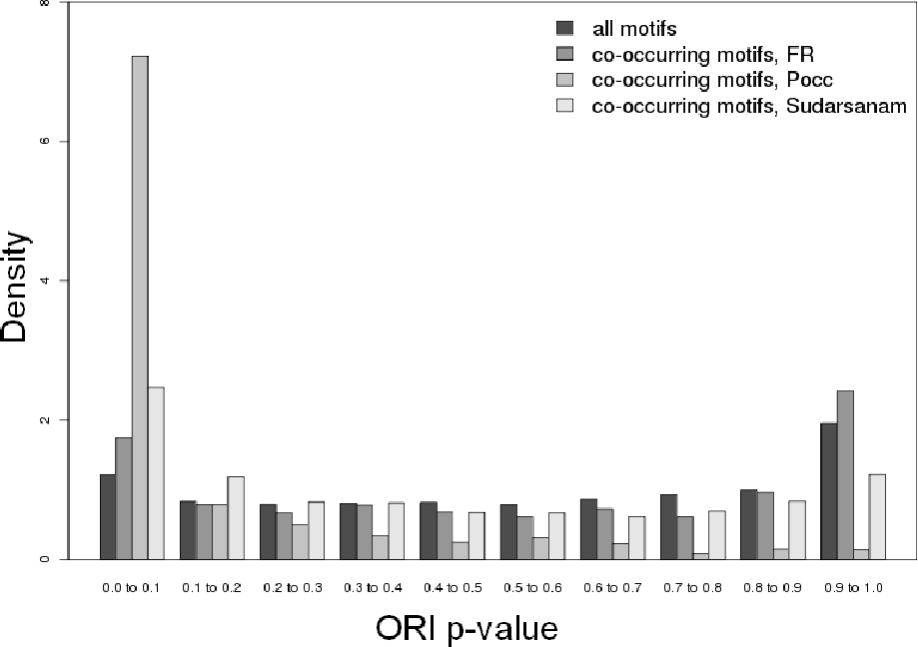
**Over-representation p-values of co-occurring motifs predicted by two approaches**. Co-occurrences based on *P_occ _*are strongly biased by PWM over-representation, while this is not the case for co-occurrences predicted using the *FR *measure. The distribution of *ORI *p-values as measure for PWM over-representation is shown 1) for all PWMs in all sets of co-regulated genes ("all motifs), 2) for the 1294 PWMs found to be significantly co-occurring with an over-represented motif according to *FR *values ("co-occurring motifs, *FR*"), 3) for the PWMs found to be co-occurring with an over-represented motif according to *P_occ _*("co-occurring motifs, *P_occ_*"), and 4) for the PWMs found to be co-occurring with an over-represented motif according to the approach of Sudarsanam et al. ("co-occurring motifs, Sudarsanam"). For the latter two approaches the 1294 pairs with the most significant co-occurrence were used.

To illustrate the difference between our approach and approaches based on statistical tests, we calculated co-occurrence p-values using the method of Yu et al. [22], and using the method of Sudarsanam et al. [18]. The approach by Yu et al. evaluates co-occurrences using two p-values, one for co-occurrences, *P_occ_*, and one for the bias in distances between pairs of sites, *P_d_*. Here we focused on *P_occ_*, the probability of observing an equal or greater number of co-occurrences, calculated based on the number of sequences in the co-regulated set versus the size of the genome-wide set, the number of co-occurrences between two motifs in the genome-wide set, and the number of co-occurrences in the co-expressed set. The approach by Sudarsanam et al. uses a cumulative hypergeometric model to evaluate the significance of the observed number of co-occurrences for a motif pair, by comparing it to the distribution of expected co-occurrences given the number of occurrences of the individual motifs. We applied our *FR *approach, the *P_occ _*approach, and the Sudarsanam approach on all sets of co-expressed genes, and compared the results in terms of the over-representation of co-occurring motifs. Fig. 3 shows that the distribution of *ORI *p-values for all 1294 PWMs co-occurring significantly with an over-represented motif is similar to that of all PWMs, confirming that the *FR *approach is not biased by motif over-representation. Indeed, the majority of predicted co-occurring motifs are not over-represented. In contrast, the distribution of *ORI *p-values of predicted co-occurring motifs in the top 1294 pairs as predicted by *P_occ_*, showed a strong bias towards lower *ORI *p-values, indicating that this method is strongly biased by motif over-representation. The fact that with increasing motif over-representation the expected number of co-occurrences modeled by the hypergeometric distribution also increases, makes the approach described by Sudarsanam et al. [18] relatively robust against the bias caused by motif over-representation, but less so than the *FR *measure. However, this method does not use a reference set of sequences during the evaluation of significance, making it the most easily affected of these three approaches by PWM-to-PWM similarities (as measured by GC content differences). A relatively high number of co-occurring pairs predicted by the approach by Sudarsanam et al., have similar GC content levels, and pairs of motifs with large differences in GC content are relatively rarely predicted to be co-occurring (Fig. S6 in Additional file 9).

As an illustration, for the set of promoters of liver- and kidney-specific genes in mouse, the top co-occurrences in terms of *P_occ _*were strongly dominated by PWM pairs containing HNF1 and HNF4, which were both strongly over-represented in this cluster. In the top 20 motif pairs, 18 involved HNF1, which was found to have significant *P_occ _*values with most other over-represented motifs, such as those for HNF4 and Ikaros. The pair HNF1 - HNF4 had the lowest *P_occ _*value (2.06e-11). However *FR(*HNF4|HNF1*)_set _*was only moderately higher than *FR(*HNF4|HNF1*)_genomic _*(1.22 vs 1.01, p-value 0.25). Indeed, only 27 out of 62 (44%) HNF4 sites co-occurred with HNF1 sites, which were present in 60 out of 155 (39%) sequences in this cluster. Even though both motifs were over-represented in this cluster, they did not have a strong tendency to be present in the same sequences. The measure described by Sudarsanam et al. too, predicted a number of significant co-occurrences involving HNF1. Strikingly, the top 10 motifs predicted to co-occur with HNF1 motifs have similarly low GC content value as the HNF1 motif (mean difference in GC content: 5.7%), while this is not the case for the 10 motifs with most significant *FR *values with regard to HNF1 (mean difference in GC content: 20.9%). Collectively, these results indicate that *P_occ _*is more related to co-over-representation than to co-occurrence, and that the measure proposed by Sudarsanam et al. is relatively sensitive to GC content similarities. Our proposed approach, on the other hand, can find non-over-represented motifs, which are likely to be missed by traditional approaches, and is less influenced by PWM-to-PWM GC content similarity.

### Identification of TFs co-occurring with NF-κB or IRF in gene sets having specific expression patterns on TLR stimulation

Several TFs such as NF-κB, IRF, and AP-1 are known to play roles in gene expression evoked by TLR signaling. However, little is known about involvement of other TFs and how these TFs orchestrate variety of gene expression patterns.

**Figure 4:**
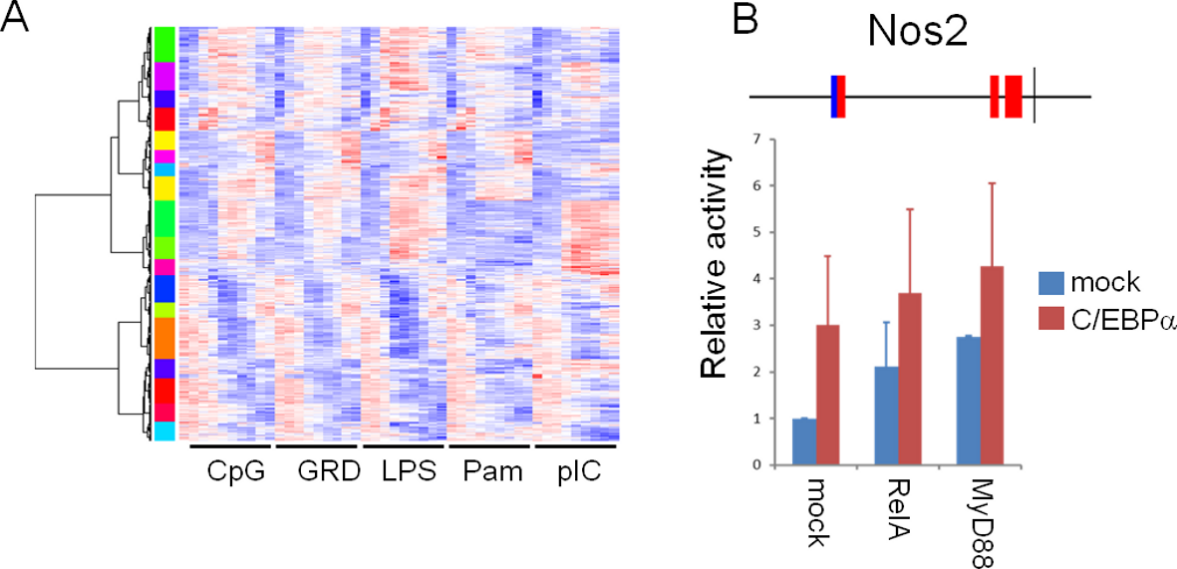
**Gene expression patterns in TLR-stimulated dendritic cells and identification of a TF pair synergistically upregulating target promoters**. (A) Hierarchical clustering of gene expression in DCs upon TLR-stimulation. A heatmap of gene expression is shown with clusters indicated by the colored banner besides the heatmap. (B) The *Nos2 *promoter was synergistically controlled by RelA or MyD88 with C/EBPα. Schematic representation of the Nos2 promoter structure is shown (red box, NF-κB sites; blue box, C/EBPα sites). The *Nos2 *promoter was cloned upstream of a luciferase encoding gene. The resulting plasmid, where luciferase is under the control of the *Nos2 *promoter, was transfected into HEK 293 cells with RelA or MyD88 and C/EBPα. After 24 hours, luciferase activity was measured as described in Methods. Error bars represent standard deviations of duplicate experiments. The data shown is a representative of three independent experiments with essentially identical results.

To elucidate the diverse gene expression patterns generated by combinations of various TFs, we next applied our method for the analysis on TLR signaling. We used a large scale microarray dataset on gene expression in bone marrow-derived DCs after stimulation with various TLR stimuli [9]. The gene expression values were calculated and hierarchically clustered into 18 clusters (Fig. 4A, and Fig. S7 in Additional file 10 for details).

Frequency ratio analysis revealed that a wide variety of TFs had significant co-occurrence with each other (Table S3 in Additional file 11). Firstly, we looked into TFs co-occurring with the binding motifs of NF-κB or IRF, since *ORI *analysis showed that these two motifs are significantly over-represented in the clusters, suggesting their key roles in TLR signaling, consistent with former studies (Table S2 in Additional file 8). Several TFs were found to have significant co-occurrence with NF-κB or IRF (Table 2). NF-κB and IRF motifs were found to co-occur significantly with themselves (homotypic co-occurrences) in clusters 1, 9, and 13. Clusters 1 and 9 contained genes whose expression levels peaked at about 0.5 to 4 hours after stimulation, suggesting multiple NF-κB sites in promoters of those genes enable rapid induction (Fig. S7A in Additional file 10). Genes in cluster 13 were specific for poly (I:C) stimulation, and their expression peaked at around 6 hours after stimulation (Fig. S7B in Additional file 10). In addition to various IRF family TFs, the PWM M00063 represents Stat1, implying that these genes may be induced secondarily by type I interferon. Other well-known motifs found were c-Fos (MA0099), presumably representing the AP-1 motif. These collectively suggest that our method could identify known TF pairs involved in TLR signaling.

**Table 2:** Overview of the co-occurrences in TLR-stimulated DC gene expression patterns.

Cluster index	Promoter set	PWM A	PWM B	TF(s) associated with PWM A	TF(s) associated with PWM B
1	nonCpG	M00054	M00054	NF-kappaB	NF-kappaB
1	all	M00054	M00054	NF-kappaB	NF-kappaB
9	all	M00054	M00054	NF-kappaB	NF-kappaB
12	all	M00063	M01171	IRF family	Bcl6
12	all	M00063	M00963	IRF family	T3R
12	nonCpG	M00063	M00963	IRF family	T3R
12	nonCpG	M00063	M01171	IRF family	Bcl6
13	all	M00063	M00063	IRF family	IRF
13	all	M00063	PB0060	IRF family	Hand1::Tcfe2a, Hand1:E47, SMAD, Smad3
13	all	M00063	M00701	IRF family	Smad3
14	all	M00054	M00249	NF-kappaB	CHOP:C/EBPalpha
14	all	M00054	M00257	NF-kappaB	RREB-1
14	all	M00054	M00769	NF-kappaB	AML, Osf2, PEBP, Runx1
18	all	M00063	MA0099	IRF family	Fos

We found that in cluster 14, CCAAT enhancer binding protein alpha (C/EBPα) had significant co-occurrence with NF-κB (Table 2, p < 6.23e-3). C/EBP family transcription factors are reported to be involved in TLR signaling-induced gene expression such as cytokine gene expression [38]. Importantly, while *P_occ _*is relatively low for the pair NF-κB - C/EBPα, up to 30 out of 199 PWMs have a *P_occ _*< 0.01 for co-occurrence with the over-represented NF-κB motif in cluster 14 (data not shown), making *P_occ _*not useful for predicting co-occurrences in this case. A gene in the cluster, *Nos2*, has C/EBP motifs and NF-κB motifs in its promoter (Fig. 4B, upper scheme), thus the promoter activity is expected to be controlled by C/EBP and/or NF-κB. To check this, we cloned the *Nos2 *promoter, and its activity upon over-expression of TFs or signaling molecules MyD88 simultaneously with C/EBPα was checked by luciferase assay. The activity of the *Nos2 *promoter was up-regulated only by over-expression of C/EBPα, RelA (a major component of NF-κB [39]), or MyD88 (an adaptor protein of TLR signaling pathways [40]) (Fig. 4B), indicating these TFs positively regulate the *Nos2 *promoter. Moreover, when C/EBPα was over-expressed simultaneously with RelA or MyD88, luciferase activity increased compared to that on RelA or MyD88 over-expression alone. This result indicated that C/EBPα controls the expression of the *Nos2 *gene, and also suggested that it controls the expression of genes other than *Nos2 *in clusters 14.

### Synergistic activation of TLR-regulated promoters by NF-κB and C/EBPα

**Figure 5:**
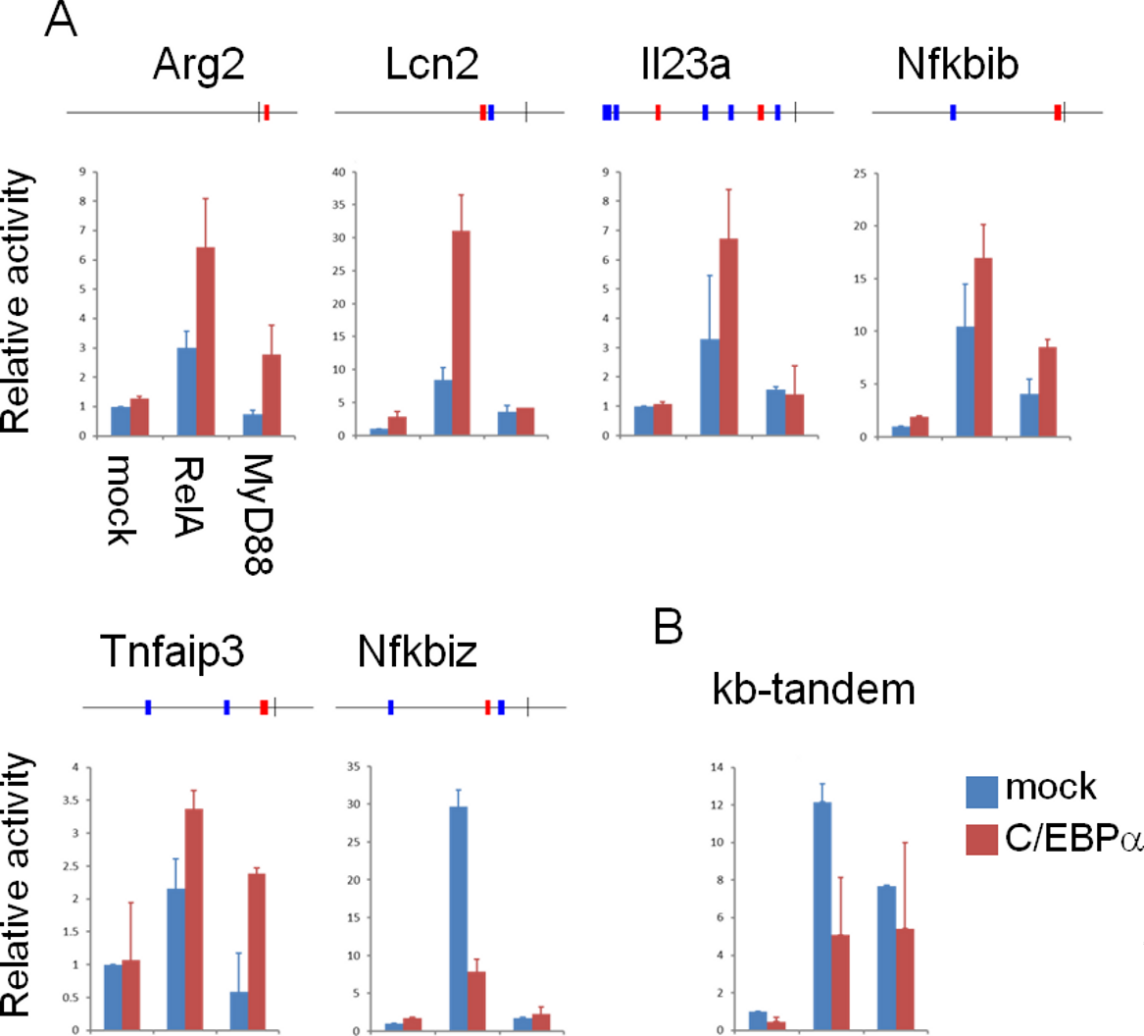
**C/EBPa regulates a set of promoters with NF-κB motif**. (A) Promoters with the co-occurrence of NF-κB and C/EBPα sites were suppressed (Nfkbiz), or activated (others) by C/EBPα. (B) κB-tandem promoter was not activated but rather suppressed by C/EBPα expression. Promoter structures are shown as in Figure 4B. Error bars represent standard deviations of duplicate experiments. All the results shown are representative of three independent experiments with essentially identical results

The above results prompted us to check if a broader array of promoters is regulated by C/EBPα. We tested whether C/EBPα controls NF-κB-regulated promoters or not. Since four PWMs in our PWM set represent C/EBP TFs (M00249, M00622, M00201, and M00159), we picked up promoters having predicted TFBSs for NF-κB and one of the four motifs (Fig. 5). We also added as a positive control Arg2, which has a predicted NF-κB site and has been reported to be a target of C/EBPβ [41].

Five promoters out of 6 tested were up-regulated synergistically by RelA and/or MyD88 and C/EBPα (Fig. 5A), whereas activation of one promoter was suppressed by C/EBPα over-expression (Nfkbiz). In contrast, the tandem-kB luciferase reporter was suppressed by C/EBPα over-expression (Fig. 5B), indicating a specific activation of promoters by C/EBPα. These results suggested that C/EBPα synergistically and specifically up-regulates the activity of a set of promoters regulated by NF-κB. Taken together, our method successfully identified a pair of transcription factors involved in the immune response.

## Conclusions

In this study, we introduced a new measure for regulatory motif co-occurrence, and investigated genome-wide co-occurrence tendencies between pairs of regulatory motifs. Our initial results show that some motif pairs have a strong tendency to co-occur, while other pairs have a strong tendency to avoid co-occurrence. However, further investigation showed that these tendencies reflect GC content fluctuations in promoter sequences, rather than a genome-wide level of combinatorial regulation: semi-artificial sequences in which GC content fluctuations were identical to real sequences showed very similar trends. On the other hand, this trend was lost in completely artificial sequences. Also, we found that only few pairs of regulatory motifs had exceptionally high or low *FR *values in the genomic set of sequences as compared to semi-artificial sequences. Pairs that did show exceptionally high *FR *values often involved the TATA-box motif, which might reflect the tendency of strictly regulated non-CpG island-associated promoters to contain a TATA-box more frequently than other promoter sequences.

In addition, we showed that the tendencies were completely different between CpG^high ^promoters and CpG^low ^promoters. Our measure for regulatory motif co-occurrences showed a relatively limited range in CpG^high ^promoters compared to CpG^low ^promoters. Similar observations were made for a set of randomly selected oligonucleotide motifs. These observations reflect a fundamental difference between these two types of promoters. CpG islands have been reported to be associated with ubiquitously expressed genes and housekeeping genes, while genes not associated with CpG islands tend to be tissue-specific or condition-specific genes [42,43]. On the promoter sequence level too, there are considerable differences: while the promoters of CpG island-associated genes tend to lack typical core promoter elements and tissue-specific TFBSs, the promoters of genes not associated with CpG islands tend to contain TATA boxes or other core promoter elements and TFBSs allowing their precise regulation of expression [44]. The apparent lack of high and low *FR *values in the genome-wide set of CpG^high ^promoters might reflect a relatively low need for complex combinatorial regulation, compared to CpG^low ^promoters. On the other hand, for tissue- or condition-specific genes combinatorial regulation might be necessary to ensure spatio-temporal specificity, reflected in the larger range of *FR *values observed in CpG^low ^promoters.

Keeping the above observations in mind, in the proposed method, for the set of promoter sequences of interest, the significance of co-occurring pairs was estimated using a random sampling procedure. This approach thus takes into account the genomic tendency of motif pairs of similar structure to appear in the same promoter sequence. Furthermore, we considered CpG^high ^promoters and CpG^low ^promoters as separate cases. A recent study on TFBS analysis has led to a similar recommendation [45]. Moreover, we excluded overlapping pairs of sites to avoid bias caused by similarity between motifs.

Applying our method to a large number of tissue-specific sets of mouse promoters, we could predict a large number of pairs of significantly co-occurring TFBS pairs. One example is the pair HNF1 - FOXP1, for which we found binding sites to be significantly co-occurring in the promoters of genes with specific expression in liver and kidney. Moreover, the TFBSs of this pair of TFs showed a tendency to be located proximally to each other, with the FOXP1 TFBSs located upstream of the HNF1 TFBSs. Importantly, our approach demonstrated improved robustness against biases caused by strongly over-represented motifs in comparison to a previously reported statistics-based method. Indeed, the majority of significant interactions we found involved motifs that were not over-represented (a considerable fraction was actually under-represented). This was also the case for C/EBPα binding sites in cluster 14 from the DC expression data. Such motifs would thus not be detected by standard over-representation analysis.

For one of the significantly co-occurring TF pairs involved in TLR signaling we could verify the predicted combinatorial regulation. We found that C/EBPα co-regulates a set of promoters with NF-κB. Co-regulation by NF-κB and C/EBP has previously been reported. Lcn2 and Arg2 were reported as targets of C/EBPβ [41], confirming that the Frequency Ratio could predict biologically meaningful TF pairs. Moreover, C/EBPα itself has recently been found to control a number of RelA-dependent inflammatory promoters, and NF-κB activation synergistically with PU.1 [46], further supporting our findings on the significance of the NF-κB-C/EBP pair. It would be interesting to check the involvement of other pairs identified as in Table 2 in TLR-induced gene expression patterns.

There is some room for possible improvements of our approach. First of all, epigenetic factors that might be responsible for tissue- or condition-specific expression should be taken into account. At present, chromatin remodeling data is still limited to a small number of cell types, which makes it difficult to incorporate in our approach. Undoubtedly, as the amount of available data increases, there will be a need to incorporate it, resulting in approaches combining both TFBS information and epigenetic information. Importantly, while we have limited our analysis to promoter sequences here, the role of distal enhancers in the regulation of transcription is generally accepted. As epigenetic data for various cell types increases, we will become able to apply our method not only to promoter regions but also to enhancers, and investigate potential differences in combinatorial regulation occurring in promoter and enhancers. Secondly, since our approach relies on TFBS prediction, which is still known to have a low specificity, further developments in the prediction of TFBSs, and additional genome-wide binding data is likely to improve our method as well as other TFBS prediction-based methods.

In conclusion, our *FR *approach circumvents biases which former methodology suffers from, and we could identify some meaningful co-occurring TFBS pairs, one of which was experimentally supported. We believe this approach can help us detect combinatorial interactions between TFs in the regulation of transcription, and we also believe that this sets a basis for future developments in computational identification of combinatorial gene regulation.

An online application of our method, which we call REgulatory MOtif COmbination Detector (REMOCOD), is available at our website [47].

## List of abbreviations used

C/EBP: CCAAT enhancer binding protein; ChIP: chromatin immunoprecipitation; DC: dendritic cell; FR: frequency ratio; ORI: over-representation index; PPR: pattern-recognition receptor; PWM: position weight matrix; RMA: robust multi-array average; TF: transcription factor; TFBS: transcription factor binding site; TLR: Toll-like receptor; TSS: transcription start site.

## Competing interests

The authors declare that they have no competing interests.

## Authors' contributions

AV and YK conceived of the study. AV performed the bioinformatics analysis, and prepared the manuscript. YK carried out experimental validations and prepared the manuscript. DMS and SA supervised the project and helped with discussion and interpretation of results and with drafting the manuscript. All authors read and approved the final manuscript.

## Supplementary Material

Additional file 1 **Figure S1 - (PPT, Powerpoint file) Workflow of our framework for the detection of co-occurring motifs**. The analysis of genome-wide tendencies starts with a set of TFBSs, predicted in promoter sequences and a set of PWMs. For each pair of motifs, *FR *values are calculated, and used for further analysis of genome-wide tendencies. The analysis of co-occurrences in sets of co-regulated genes similarly starts with the prediction of TFBSs. Using these, significantly over-represented TFBSs are detected, and for each motif the tendency to co-occur with each of the over-represented motifs is analysed. The significance of the co-occurrences is evaluated using a random sampling approach, sampling sequences from the genomic set of promoters. Click here for file

Additional file 2 **Supporting text - (DOC, Word file) On the asymmetry of the Frequency Ration measure**. Click here for file

Additional file 3 **Figure S2 - (PPT, Powerpoint file) Genome-wide tendencies of Frequency Ratios in human promoter sequences**. (A) Histogram of *FR *values for all PWM pairs in the genomic set of human promoter sequences. (B,C,D) Plots of GC content differences as measure of PWM-to-PWM dissimilarity (Y-axis) versus *FR *values (X-axis, same as in A), for all promoters (B), CpG^high ^promoters (C), and CpG^low ^promoters (D). Click here for file

Additional file 4 **Figure S3 - (PPT, Powerpoint file) Tendencies of Frequency Ratio in semi-artificial and completely artificial sequences**. Plot of GC content differences as measure of PWM-to-PWM dissimilarity (Y-axis) versus *FR *values (X-axis) in semi-artificial sequences (A), and completely artificial sequences (B), semi-artificial CpG^high ^sequences (C), and semi-artificial CpG^low ^sequences (D). Click here for file

Additional file 5 **Figure S4 - (PPT, Powerpoint file) Genome-wide tendencies of Frequency Ratios for 200 randomly selected 7-mers in human and mouse promoter sequences**. Plots of GC content differences (Y-axis) versus *FR *values (X-axis) are shown for all human promoters (A), all mouse promoters (B), human CpG^high ^promoters (C), mouse CpG^high ^promoters (D), human CpG^low ^promoters (E), and mouse CpG^low ^promoters (F). Click here for file

Additional file 6 **Figure S5 - (PPT, Powerpoint file) Heatmap representation of the average expression values for each of the 44 clusters obtained from the GNF GeneAtlas mouse data**. Click here for file

Additional file 7 **Table S1 - (XLS, Excel Spreadsheet) Summary of main tissues for the 44 clusters obtained from the GNF GeneAtlas data**. Click here for file

Additional file 8 Table S2 - (XLS, Excel Spreadsheet) Summary of over-represented PWM motifs in tissue-specific sets of mouse promoters (GNF GeneAtlas data and Amit et al. data) Click here for file

Additional file 9 **Figure S6 - (PPT, Powerpoint file) Histogram of the PWM-to-PWM GC content differences of co-occurring motifs predicted by three approaches**. Co-occurrences predicted by the *FR *measure are least affected by PWM-to-PWM GC content differences. The distribution of GC content differences of predicted co-occurring pairs of PWMs is shown 1) for the 1294 PWMs found to be significantly co-occurring with an over-represented motif according to *FR *values ("co-occurring motifs, *FR*"), 2) for the PWMs found to be co-occurring with an over-represented motif according to *P_occ _*("co-occurring motifs, *P_occ_*"), and 3) for the PWMs found to be co-occurring with an over-represented motif according to the approach of Sudarsanam et al. ("co-occurring motifs, Sudarsanam"). For the latter two approaches the 1294 pairs with the most significant co-occurrence were used. Click here for file

Additional file 10 **Figure S7 - (PPT, Powerpoint file) Heatmap representation of clusters of TLR-stimulated DC gene expression data referred to in the main text**. Click here for file

Additional file 11 **Table S3 - (XLS, Excel Spreadsheet) Summary for the co-occurrences in tissue-specific sets of mouse promoters (GNF GeneAtlas data and Amit et al. data)**. Click here for file

## References

[B1] Arnone MI Davidson EH The hardwiring of development: organization and function of genomic regulatory systems Development 1997 124 1851 1864 9169833 10.1242/dev.124.10.1851

[B2] Levine M Tjian R Transcription regulation and animal diversity Nature 2003 424 147 151 10.1038/nature01763 12853946

[B3] Odom DT Dowell RD Jacobsen ES Nekludova L Rolfe PA Danford TW Gifford DK Fraenkel E Bell GI Young RA Core transcriptional regulatory circuitry in human hepatocytes Mol Syst Biol 2006 10.1038/msb4100059 PMC1681491 16738562

[B4] Inoue T Wang MQ Ririe TO Fernandes JS Sternberg PW Transcriptional network underlying Caenorhabditis elegans vulval development P Natl Acad Sci USA 2005 102 4972 4977 10.1073/pnas.0408122102 PMC555976 15749820

[B5] Davidson EH Rast JP Oliveri P Ransick A Calestani C Yuh CH Minokawa T Amore G Hinman V Arenas-Mena C A genomic regulatory network for development Science 2002 295 1669 1678 10.1126/science.1069883 11872831

[B6] Zinzen RP Girardot C Gagneur J Braun M Furlong EEM Combinatorial binding predicts spatio-temporal cis-regulatory activity Nature 2009 462 65 U72 10.1038/nature08531 19890324

[B7] Ravasi T Suzuki H Cannistraci CV Katayama S Bajic VB Tan K Akalin A Schmeier S Kanamori-Katayama M Bertin N An atlas of combinatorial transcriptional regulation in mouse and man Cell 2010 140 744 752 10.1016/j.cell.2010.01.044 20211142 PMC2836267

[B8] Ramsey SA Klemm SL Zak DE Kennedy KA Thorsson V Li B Gilchrist M Gold ES Johnson CD Litvak V Uncovering a macrophage transcriptional program by integrating evidence from motif scanning and expression dynamics Plos Comput Biol 2008 4 10.1371/journal.pcbi.1000021 PMC2265556 18369420

[B9] Amit I Garber M Chevrier N Leite AP Donner Y Eisenhaure T Guttman M Grenier JK Li WB Zuk O Unbiased reconstruction of a mammalian transcriptional network mediating pathogen responses Science 2009 326 257 263 10.1126/science.1179050 19729616 PMC2879337

[B10] Wang Y Zhang XS Xia Y Predicting eukaryotic transcriptional cooperativity by Bayesian network integration of genome-wide data Nucleic Acids Res 2009 37 5943 5958 10.1093/nar/gkp625 19661283 PMC2764433

[B11] Aguilar D Oliva B Topological comparison of methods for predicting transcriptional cooperativity in yeast Bmc Genomics 2008 9 10.1186/1471-2164-9-137 PMC2315657 18366726

[B12] Pilpel Y Sudarsanam P Church GM Identifying regulatory networks by combinatorial analysis of promoter elements Nat Genet 2001 29 153 159 10.1038/ng724 11547334

[B13] Banerjee N Zhang MQ Identifying cooperativity among transcription factors controlling the cell cycle in yeast Nucleic Acids Research 2003 31 7024 7031 10.1093/nar/gkg894 14627835 PMC290262

[B14] Kato M Hata N Banerjee N Futcher B Zhang MQ Identifying combinatorial regulation of transcription factors and binding motifs Genome Biol 2004 5 10.1186/gb-2004-5-8-r56 PMC507881 15287978

[B15] Wang JB A new framework for identifying combinatorial regulation of transcription factors: A case study of the yeast cell cycle J Biomed Inform 2007 40 707 725 10.1016/j.jbi.2007.02.003 17418646

[B16] Datta D Zhao HY Statistical methods to infer cooperative binding among transcription factors in Saccharomyces cerevisiae Bioinformatics 2008 24 545 552 10.1093/bioinformatics/btm523 17989095

[B17] Murakami K Imanishi T Gojobori T Nakai K Two different classes of co-occurring motif pairs found by a novel visualization method in human promoter regions Bmc Genomics 2008 9 10.1186/1471-2164-9-112 PMC2292176 18312685

[B18] Sudarsanam P Pilpel Y Church GM Genome-wide co-occurrence of promoter elements reveals a cis-regulatory cassette of rRNA transcription motifs in Saccharomyces cerevisiae Genome Research 2002 12 1723 1731 10.1101/gr.301202 12421759 PMC187556

[B19] Hannenhalli S Levy S Predicting transcription factor synergism Nucleic Acids Research 2002 30 4278 4284 10.1093/nar/gkf535 12364607 PMC140535

[B20] Gertz J Siggia ED Cohen BA Analysis of combinatorial cis-regulation in synthetic and genomic promoters Nature 2009 457 215 U113 10.1038/nature07521 19029883 PMC2677908

[B21] Lee D Karchin R Beer MA Discriminative prediction of mammalian enhancers from DNA sequence Genome Research 2011 21 2167 2180 10.1101/gr.121905.111 21875935 PMC3227105

[B22] Yu XP Lin J Masuda T Esumi N Zack DJ Qian J Genome-wide prediction and characterization of interactions between transcription factors in Saccharomyces cerevisiae Nucleic Acids Research 2006 34 917 927 10.1093/nar/gkj487 16464824 PMC1361616

[B23] Yu X Lin J Zack DJ Qian J Identification of tissue-specific cis-regulatory modules based on interactions between transcription factors BMC Bioinformatics 2007 8 437 10.1186/1471-2105-8-437 17996093 PMC2194798

[B24] Yamashita R Wakaguri H Sugano S Suzuki Y Nakai K DBTSS provides a tissue specific dynamic view of Transcription Start Sites Nucleic Acids Res 2010 38 D98 D104 10.1093/nar/gkp1017 19910371 PMC2808897

[B25] Carninci P Kasukawa T Katayama S Gough J Frith MC Maeda N Oyama R Ravasi T Lenhard B Wells C The transcriptional landscape of the mammalian genome Science 2005 309 1559 1563 16141072 10.1126/science.1112014

[B26] Rhead B Karolchik D Kuhn RM Hinrichs AS Zweig AS Fujita PA Diekhans M Smith KE Rosenbloom KR Raney BJ The UCSC Genome Browser database: update 2010 Nucleic Acids Research 2010 38 D613 619 10.1093/nar/gkp939 19906737 PMC2808870

[B27] Vandenbon A Nakai K Modeling tissue-specific structural patterns in human and mouse promoters Nucleic Acids Research 2010 38 17 25 10.1093/nar/gkp866 19850720 PMC2800225

[B28] Matys V Kel-Margoulis OV Fricke E Liebich I Land S Barre-Dirrie A Reuter I Chekmenev D Krull M Hornischer K TRANSFAC and its module TRANSCompel: transcriptional gene regulation in eukaryotes Nucleic Acids Res 2006 34 D108 110 10.1093/nar/gkj143 16381825 PMC1347505

[B29] Bryne JC Valen E Tang MHE Marstrand T Winther O da Piedade I Krogh A Lenhard B Sandelin A JASPAR, the open access database of transcription factor-binding profiles: new content and tools in the 2008 update Nucleic Acids Research 2008 36 D102 D106 10.1093/nar/gkn449 18006571 PMC2238834

[B30] Gupta S Stamatoyannopoulos JA Bailey TL Noble WS Quantifying similarity between motifs Genome Biol 2007 8 R24 10.1186/gb-2007-8-2-r24 17324271 PMC1852410

[B31] Su AI Wiltshire T Batalov S Lapp H Ching KA Block D Zhang J Soden R Hayakawa M Kreiman G A gene atlas of the mouse and human protein-encoding transcriptomes Proc Natl Acad Sci USA 2004 101 6062 6067 10.1073/pnas.0400782101 15075390 PMC395923

[B32] Gardinergarden M Frommer M Cpg islands in vertebrate genomes Journal of Molecular Biology 1987 196 261 282 10.1016/0022-2836(87)90689-9 3656447

[B33] Bajic VB Choudhary V Hock CK Content analysis of the core promoter region of human genes In Silico Biol 2004 4 109 125 15089758

[B34] Cereghini S Liver-enriched transcription factors and hepatocyte differentiation Faseb J 1996 10 267 282 8641560

[B35] Ktistaki E Talianidis I Modulation of hepatic gene expression by hepatocyte nuclear factor 1 Science 1997 277 109 112 10.1126/science.277.5322.109 9204893

[B36] Hayhurst GP Lee YH Lambert G Ward JM Gonzalez FJ Hepatocyte nuclear factor 4alpha (nuclear receptor 2A1) is essential for maintenance of hepatic gene expression and lipid homeostasis Mol Cell Biol 2001 21 1393 1403 10.1128/MCB.21.4.1393-1403.2001 11158324 PMC99591

[B37] Odom DT Zizlsperger N Gordon DB Bell GW Rinaldi NJ Murray HL Volkert TL Schreiber J Rolfe PA Gifford DK Control of pancreas and liver gene expression by HNF transcription factors Science 2004 303 1378 1381 10.1126/science.1089769 14988562 PMC3012624

[B38] Nerlov C The C/EBP family of transcription factors: a paradigm for interaction between gene expression and proliferation control Trends Cell Biol 2007 17 318 324 10.1016/j.tcb.2007.07.004 17658261

[B39] Hayden MS Ghosh S NF-kappa B, the first quarter-century: remarkable progress and outstanding questions Gene Dev 2012 26 203 234 10.1101/gad.183434.111 22302935 PMC3278889

[B40] Medzhitov R Preston-Hurlburt P Kopp E Stadlen A Chen CQ Ghosh S Janeway CA MyD88 is an adaptor protein in the hToll/IL-1 receptor family signaling pathways Mol Cell 1998 2 253 258 10.1016/S1097-2765(00)80136-7 9734363

[B41] Yamamoto M Uematsu S Okamoto T Matsuura Y Sato S Kumar H Satoh T Saitoh T Takeda K Ishii KJ Enhanced TLR-mediated NF-IL6-dependent gene expression by Trib1 deficiency J Exp Med 2007 204 2233 2239 10.1084/jem.20070183 17724128 PMC2118688

[B42] Smale ST Kadonaga JT The RNA polymerase II core promoter Annu Rev Biochem 2003 72 449 479 10.1146/annurev.biochem.72.121801.161520 12651739

[B43] Sandelin A Carninci P Lenhard B Ponjavic J Hayashizaki Y Hume DA Mammalian RNA polymerase II core promoters: insights from genome-wide studies Nat Rev Genet 2007 8 424 436 17486122 10.1038/nrg2026

[B44] Carninci P Sandelin A Lenhard B Katayama S Shimokawa K Ponjavic J Semple CA Taylor MS Engstrom PG Frith MC Genome-wide analysis of mammalian promoter architecture and evolution Nat Genet 2006 38 626 635 10.1038/ng1789 16645617

[B45] Roider HG Lenhard B Kanhere A Haas SA Vingron M CpG-depleted promoters harbor tissue-specific transcription factor binding signals-implications for motif overrepresentation analyses Nucleic Acids Res 2009 37 6305 6315 10.1093/nar/gkp682 19736212 PMC2770660

[B46] Jin FL Li Y Ren B Natarajan R PU.1 and C/EBP alpha synergistically program distinct response to NF-kappa B activation through establishing monocyte specific enhancers P Natl Acad Sci USA 2011 108 5290 5295 10.1073/pnas.1017214108 PMC3069155 21402921

[B47] REgulatory MOtif COmbination Detector http://sysimm.ifrec.osaka-u.ac.jp/tfbs/remocod/

