# Supplementary material for: A novel unbiased measure for motif co-occurrence predicts combinatorial regulation of transcription
Source: BMC Genomics. 2012 Dec 7;13(Suppl 7):S11. doi: 10.1186/1471-2164-13-S7-S11 (PMC3521209; doi:10.1186/1471-2164-13-S7-S11)
Supplement: Additional file 1 — Figure S1 - (PPT, Powerpoint file) Workflow of our framework for the detection of co-occurring motifs. The analysis of genome-wide tendencies starts with a set of TFBSs, predicted in promoter sequences and a set of PWMs. For each pair of motifs, FR values are calculated, and used for further analysis of genome-wide tendencies. The analysis of co-occurrences in sets of co-regulated genes similarly starts with the prediction of TFBSs. Using these, significantly over-represented TFBSs are detected, and for each motif the tendency to co-occur with each of the over-represented motifs is analysed. The significance of the co-occurrences is evaluated using a random sampling approach, sampling sequences from the genomic set of promoters. [file 1471-2164-13-S7-S11-S1.ppt]

## Slide 1
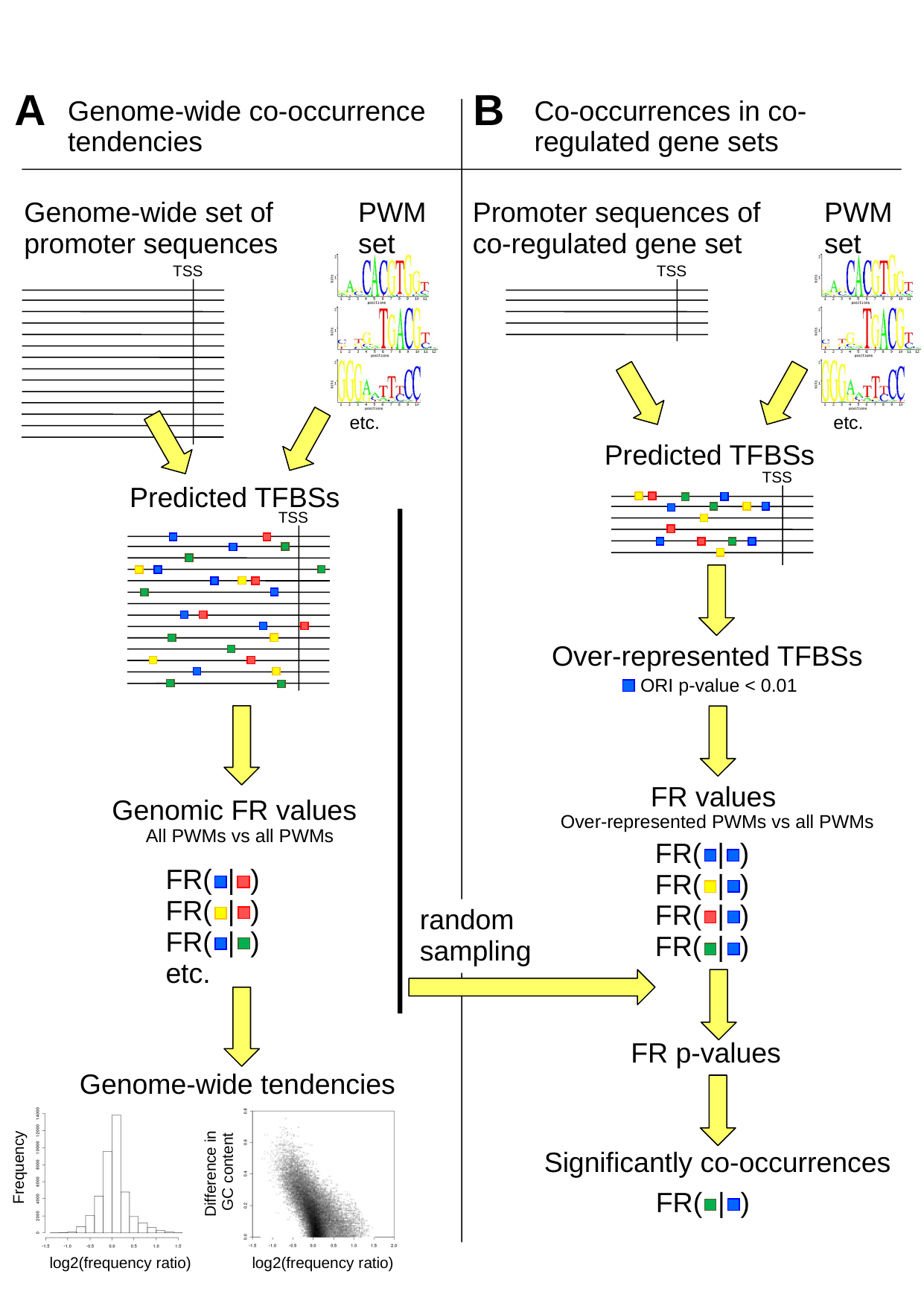

A
B
Genome-wide co-occurrence tendencies
Co-occurrences in co-regulated gene sets
Genome-wide set of promoter sequences
PWM set
Promoter sequences of co-regulated gene set
PWM set
TSS
TSS
etc.
etc.
Predicted TFBSs
TSS
Predicted TFBSs
TSS
Over-represented TFBSs
ORI p-value < 0.01
FR values
Genomic FR values
Over-represented PWMs vs all PWMs
All PWMs vs all PWMs
FR( | )
FR( | )
FR( | )
FR( | )
FR( | )
FR( | )
FR( | )
etc.
random sampling
FR p-values
Genome-wide tendencies
Significantly co-occurrences
Difference in
GC content
Frequency
FR( | )
log2(frequency ratio)
log2(frequency ratio)
