# Supplementary material for: A novel unbiased measure for motif co-occurrence predicts combinatorial regulation of transcription
Source: BMC Genomics. 2012 Dec 7;13(Suppl 7):S11. doi: 10.1186/1471-2164-13-S7-S11 (PMC3521209; doi:10.1186/1471-2164-13-S7-S11)
Supplement: Additional file 2 — Supporting text - (DOC, Word file) On the asymmetry of the Frequency Ration measure. [file 1471-2164-13-S7-S11-S2.doc]

## **Additional text supporting “A novel unbiased measure for motif co-occurrence predicts combinatorial regulation of transcription”: On the asymmetry of the Frequency Ration measure**

As mentioned in the main text of this study, our proposed measure for motif co-occurrence, the frequency ratio (*FR*) is not a symmetric measure (see main text, Methods section and Results and Disucssion section). The *FR(B|A)* value, a measure for the tendency of a motif *B* to co-occur with a motif *A*, is not necessarily the same as, or similar to, *FR(A|B)*, which is a measure for the tendency of motif *A* to co-occur with motif *B*.

However, when we compared complementary *FR* values ( *FR(A|B)* vs *FR(B|A)* ) for all position weigh matrix pairs in the genome-wide set of mouse promoters (see Fig. 1 below), we observed that, in general, the difference between *FR(A|B)* and *FR(B|A)* is small. There is a high correlation between complementary *FR* pairs, and no pairs were observed where one *FR* value is high while the complementary *FR* value is low. Similar tendencies were observed in the genome-wide set of mouse CpG^high^ promoters (Fig. 2) and CpG^low^ promoters (Fig. 3), and for human promoters (data not shown). This, we feel, fits with our notion that the genome-wide variation in *FR* values is mainly a result of sequence variation (see main text).

Nevertheless, although such examples were not observed in the genome-wide set of promoters, smaller sets of functionally related sequences might contain pairs of regulatory motifs where *FR(A|B)* is high and *FR(B|A)* is low. These might indicate that *B* does not rely on the presence of *A* for its function, while *A* does have a necessity to co-occur with *B*. This might be of interest when looking at cell line-specific enhancer regions, for example, where a so-called master regulator binds to sequences together with a variety of secondary regulators, which are dependent on this master regulator. On the other hand, in cases where both *FR(A|B)* and *FR(B|A)* are high, we can assume that TF A and TF B are mutually dependent on each other.


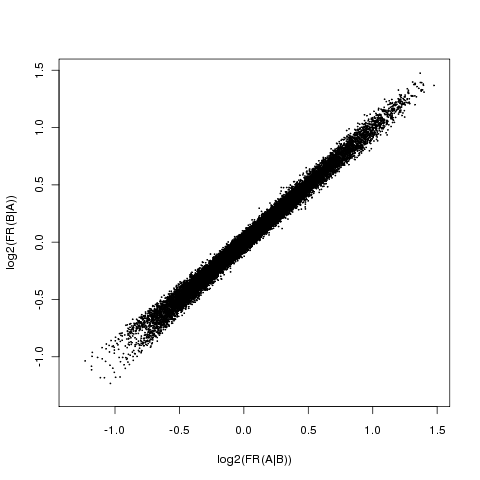


Fig. 1: Comparison of complementary *FR* values in the genome-wide set of mouse promoters. Note that both axes are in log scale.


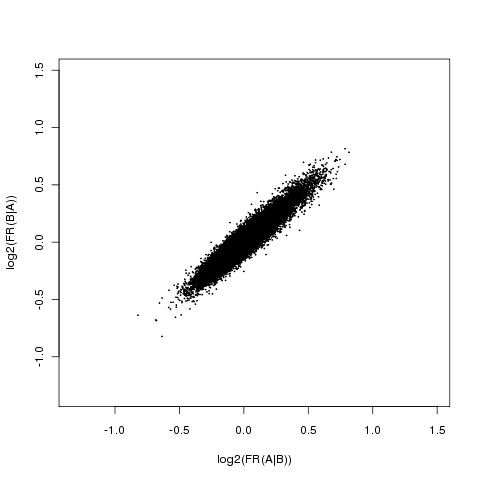


Fig. 2: Comparison of complementary *FR* values in the genome-wide set of mouse CpG^high^ promoters. Note that both axes are in log scale.


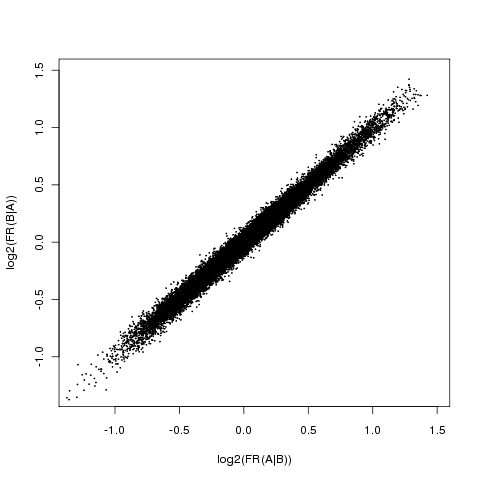


Fig. 3: Comparison of complementary *FR* values in the genome-wide set of mouse CpG^low^ promoters. Note that both axes are in log scale.
