# Supplementary material for: A novel unbiased measure for motif co-occurrence predicts combinatorial regulation of transcription
Source: BMC Genomics. 2012 Dec 7;13(Suppl 7):S11. doi: 10.1186/1471-2164-13-S7-S11 (PMC3521209; doi:10.1186/1471-2164-13-S7-S11)
Supplement: Additional file 3 — Figure S2 - (PPT, Powerpoint file) Genome-wide tendencies of Frequency Ratios in human promoter sequences. (A) Histogram of FR values for all PWM pairs in the genomic set of human promoter sequences. (B,C,D) Plots of GC content differences as measure of PWM-to-PWM dissimilarity (Y-axis) versus FR values (X-axis, same as in A), for all promoters (B), CpGhigh promoters (C), and CpGlow promoters (D). [file 1471-2164-13-S7-S11-S3.ppt]

## Slide 1
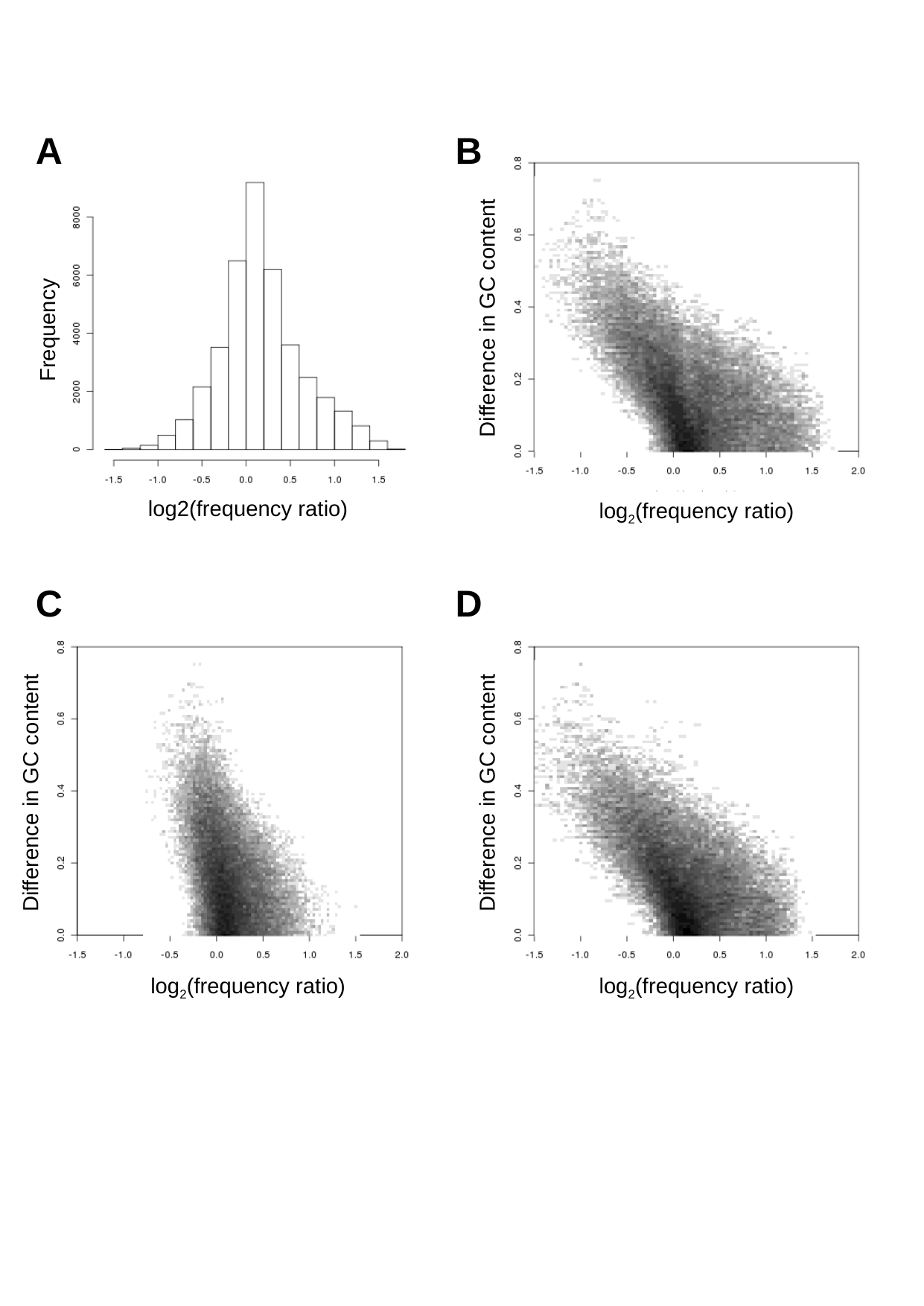

A
B
Difference in GC content
Frequency
log2(frequency ratio)
log2(frequency ratio)
C
D
Difference in GC content
Difference in GC content
log2(frequency ratio)
log2(frequency ratio)
