# Supplementary material for: A novel unbiased measure for motif co-occurrence predicts combinatorial regulation of transcription
Source: BMC Genomics. 2012 Dec 7;13(Suppl 7):S11. doi: 10.1186/1471-2164-13-S7-S11 (PMC3521209; doi:10.1186/1471-2164-13-S7-S11)
Supplement: Additional file 4 — Figure S3 - (PPT, Powerpoint file) Tendencies of Frequency Ratio in semi-artificial and completely artificial sequences. Plot of GC content differences as measure of PWM-to-PWM dissimilarity (Y-axis) versus FR values (X-axis) in semi-artificial sequences (A), and completely artificial sequences (B), semi-artificial CpGhigh sequences (C), and semi-artificial CpGlow sequences (D). [file 1471-2164-13-S7-S11-S4.ppt]

## Slide 1
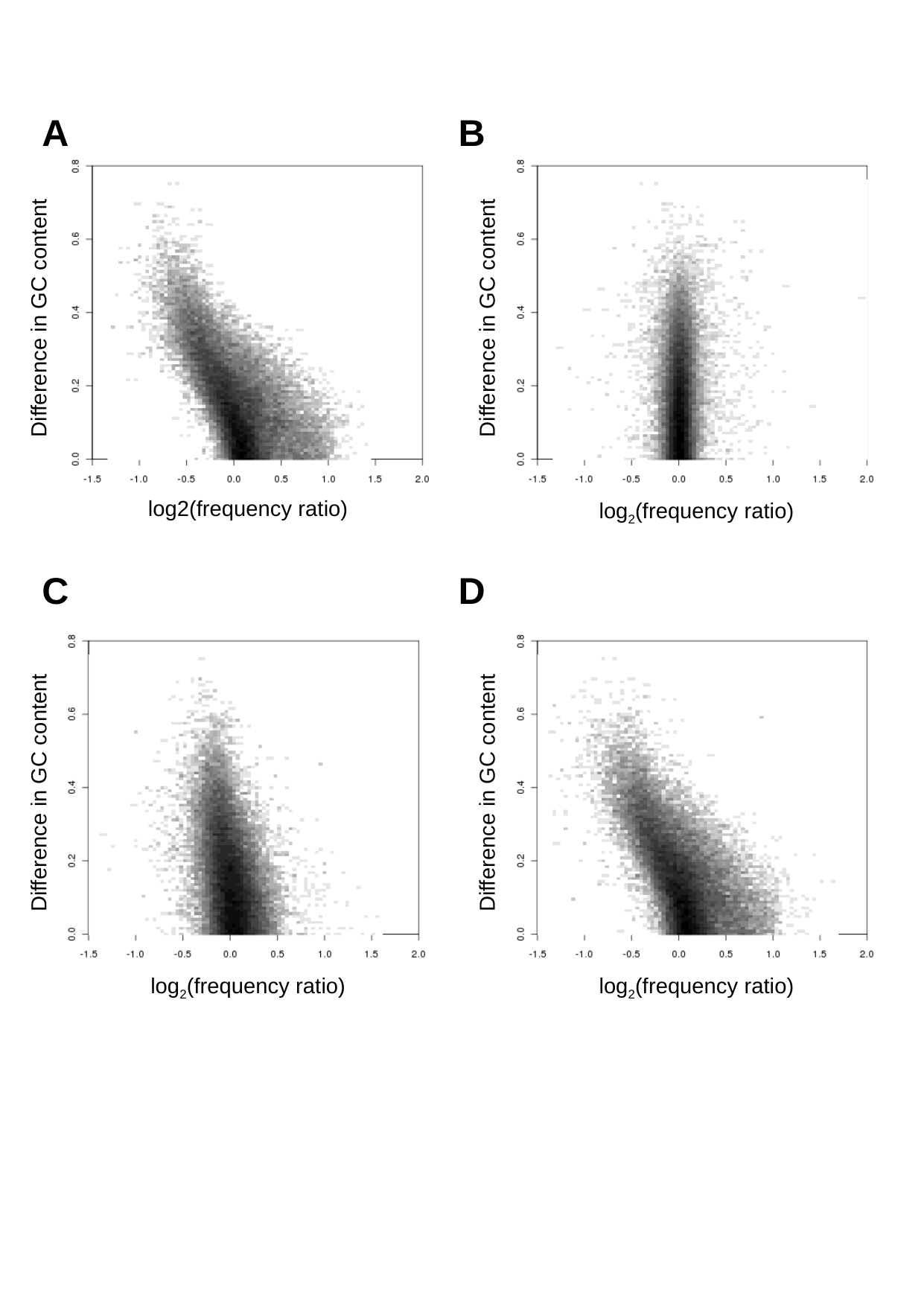

A
B
Difference in GC content
Difference in GC content
log2(frequency ratio)
log2(frequency ratio)
C
D
Difference in GC content
Difference in GC content
log2(frequency ratio)
log2(frequency ratio)
