# Supplementary material for: A novel unbiased measure for motif co-occurrence predicts combinatorial regulation of transcription
Source: BMC Genomics. 2012 Dec 7;13(Suppl 7):S11. doi: 10.1186/1471-2164-13-S7-S11 (PMC3521209; doi:10.1186/1471-2164-13-S7-S11)
Supplement: Additional file 5 — Figure S4 - (PPT, Powerpoint file) Genome-wide tendencies of Frequency Ratios for 200 randomly selected 7-mers in human and mouse promoter sequences. Plots of GC content differences (Y-axis) versus FR values (X-axis) are shown for all human promoters (A), all mouse promoters (B), human CpGhigh promoters (C), mouse CpGhigh promoters (D), human CpGlow promoters (E), and mouse CpGlow promoters (F). [file 1471-2164-13-S7-S11-S5.ppt]

## Slide 1
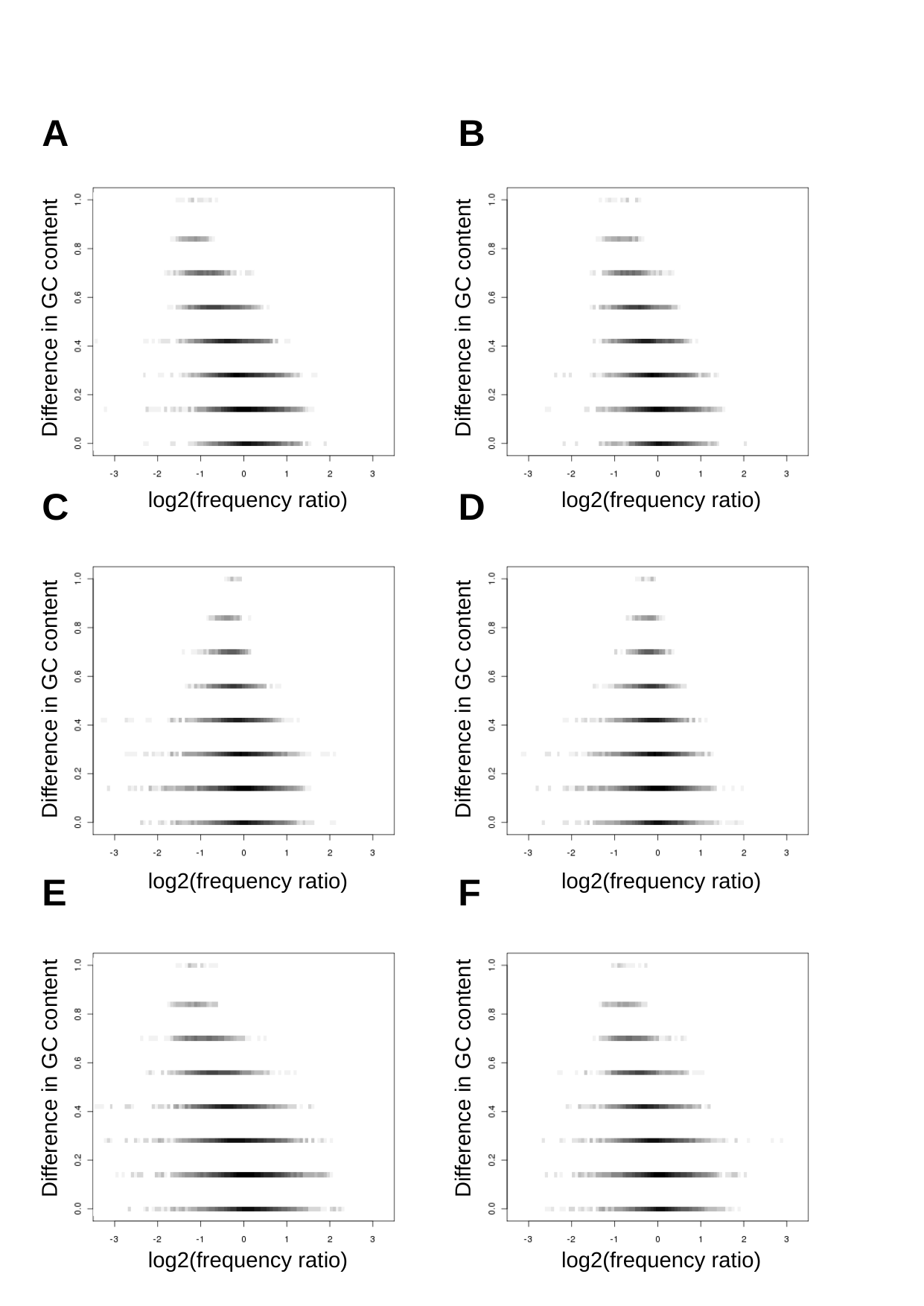

A
B
Difference in GC content
Difference in GC content
C
D
log2(frequency ratio)
log2(frequency ratio)
Difference in GC content
Difference in GC content
log2(frequency ratio)
log2(frequency ratio)
E
F
Difference in GC content
Difference in GC content
log2(frequency ratio)
log2(frequency ratio)
