# Supplementary figures and images for: A novel unbiased measure for motif co-occurrence predicts combinatorial regulation of transcription
Source: BMC Genomics. 2012 Dec 7;13(Suppl 7):S11. doi: 10.1186/1471-2164-13-S7-S11 (PMC3521209; doi:10.1186/1471-2164-13-S7-S11)

## Slide 1
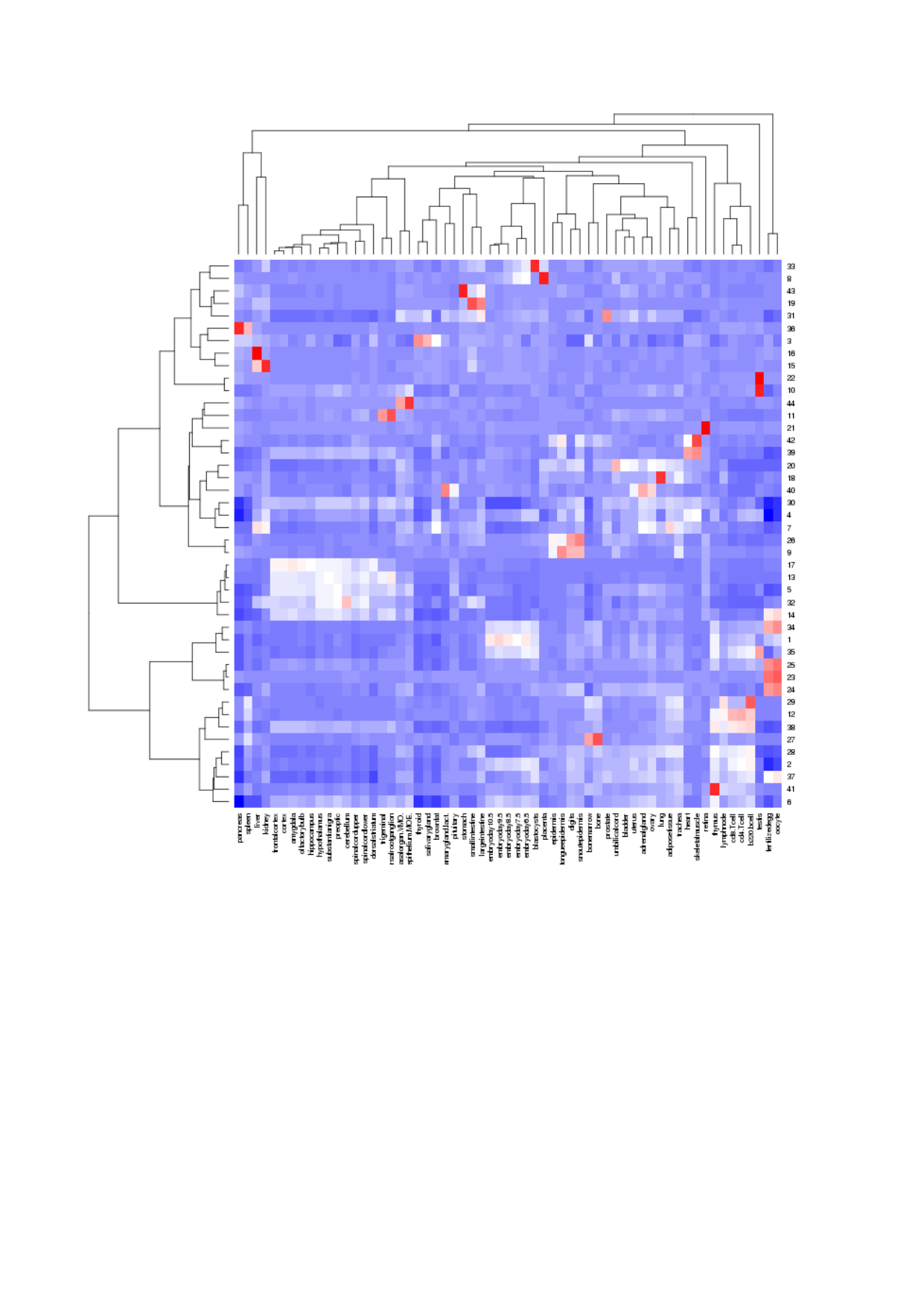

Supplement: Additional file 6 — Figure S5 - (PPT, Powerpoint file) Heatmap representation of the average expression values for each of the 44 clusters obtained from the GNF GeneAtlas mouse data. [file 1471-2164-13-S7-S11-S6.ppt]

## Slide 1
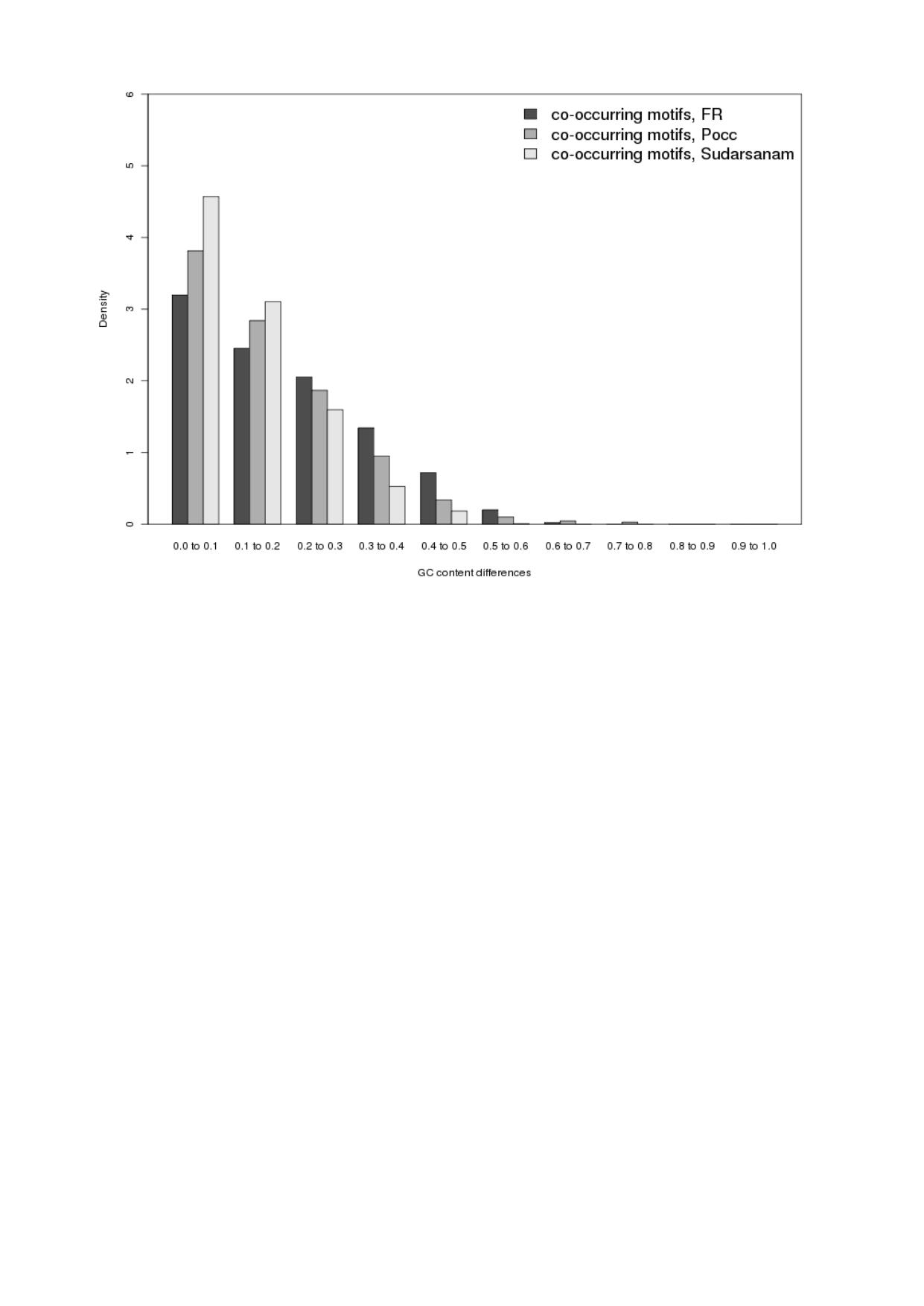

Supplement: Additional file 9 — Figure S6 - (PPT, Powerpoint file) Histogram of the PWM-to-PWM GC content differences of co-occurring motifs predicted by three approaches. Co-occurrences predicted by the FR measure are least affected by PWM-to-PWM GC content differences. The distribution of GC content differences of predicted co-occurring pairs of PWMs is shown 1) for the 1294 PWMs found to be significantly co-occurring with an over-represented motif according to FR values ("co-occurring motifs, FR"), 2) for the PWMs found to be co-occurring with an over-represented motif according to Pocc ("co-occurring motifs, Pocc"), and 3) for the PWMs found to be co-occurring with an over-represented motif according to the approach of Sudarsanam et al. ("co-occurring motifs, Sudarsanam"). For the latter two approaches the 1294 pairs with the most significant co-occurrence were used. [file 1471-2164-13-S7-S11-S9.ppt]
