# Supplementary material for: A novel unbiased measure for motif co-occurrence predicts combinatorial regulation of transcription
Source: BMC Genomics. 2012 Dec 7;13(Suppl 7):S11. doi: 10.1186/1471-2164-13-S7-S11 (PMC3521209; doi:10.1186/1471-2164-13-S7-S11)
Supplement: Additional file 10 — Figure S7 - (PPT, Powerpoint file) Heatmap representation of clusters of TLR-stimulated DC gene expression data referred to in the main text. [file 1471-2164-13-S7-S11-S10.ppt]

## Slide 1
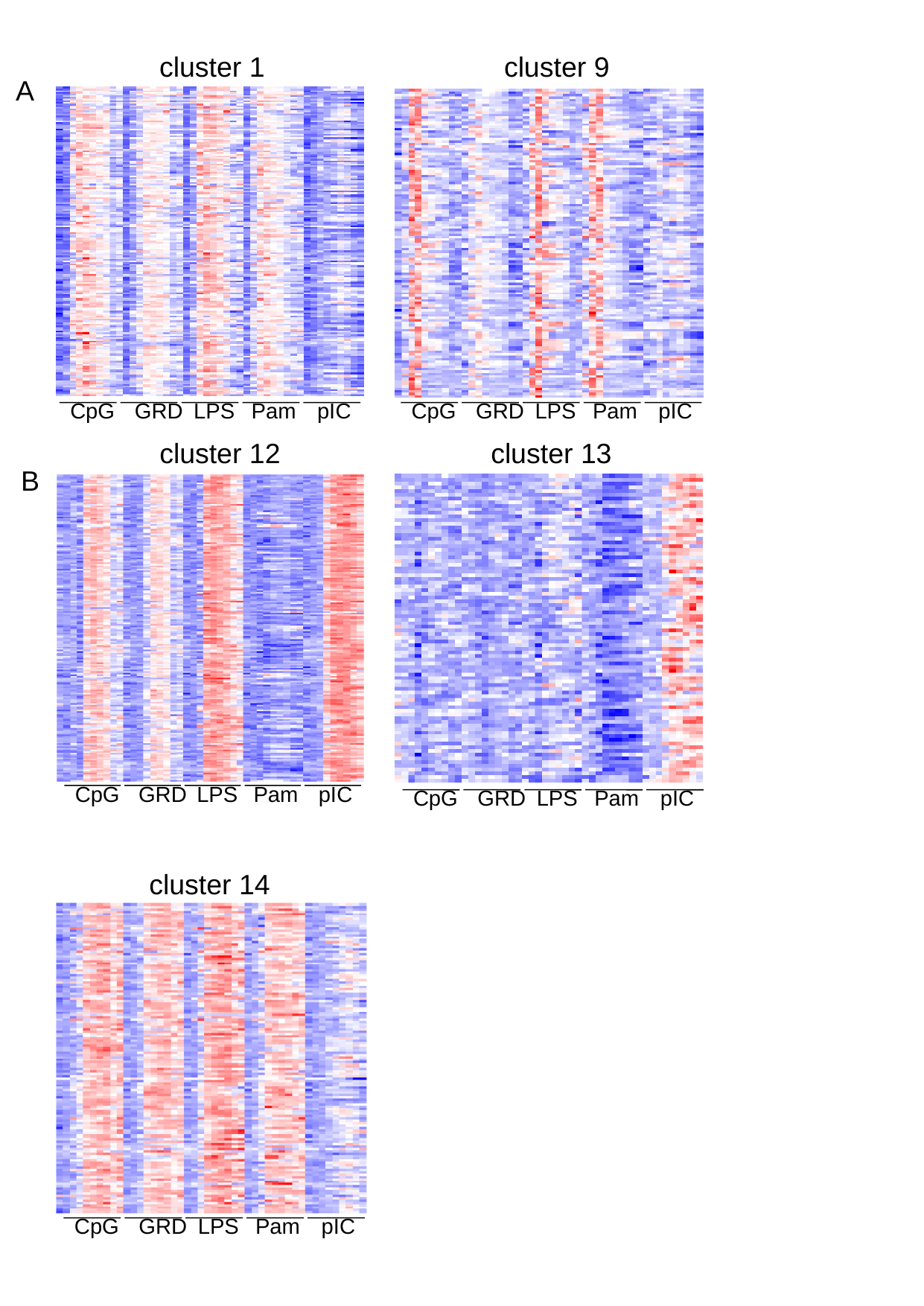

cluster 1
cluster 9
A
CpG
GRD
LPS
Pam
pIC
CpG
GRD
LPS
Pam
pIC
cluster 12
cluster 13
B
CpG
GRD
LPS
Pam
pIC
CpG
GRD
LPS
Pam
pIC
cluster 14
CpG
GRD
LPS
Pam
pIC
